# *Lactobacillus johnsonii* Generates Cyclo(pro‐trp) and Promotes Intestinal Ca^2+^ Absorption to Alleviate CKD–SHPT

**DOI:** 10.1002/advs.202414678

**Published:** 2025-01-31

**Authors:** Xiong Zeng, Lihua Sun, Huichao Xie, Shenhai Gong, Caibao Lu, Zhongwei Xu, Haidi Guan, Ben Han, Wei Wang, Zhengmin Zhang, Jieying Zhou, Shuai Wang, Yihui Chen, Weidong Xiao

**Affiliations:** ^1^ Department of General Surgery Xinqiao Hospital Army Medical University (Third Military Medical University) Chongqing 400037 China; ^2^ School of Traditional Chinese Medicine Southern Medical University Guangzhou 510515 China; ^3^ Department of Nephrology Xinqiao Hospital Army Medical University Chongqing 400037 China; ^4^ Department of Nutrition Xinqiao Hospital Army Medical University (Third Military Medical University) Chongqing 400037 China

**Keywords:** chronic kidney disease, secondary hyperparathyroidism, *Lactobacillus johnsonii*, cyclo(pro‐trp), intestinal epithelial cells, Ca^2+^

## Abstract

Patients with chronic kidney disease (CKD) are at a high risk of developing secondary hyperparathyroidism (SHPT), which may cause organ dysfunction and increase patient mortality. The main clinical interventions for CKD–SHPT involve calcium supplements to boost absorption, but ineffective for some patients, and the reasons remain unclear. Here, CKD mice are divided into high and low groups based on intact parathyroid hormone (iPTH) levels. The high group exhibits significant changes in gut microbes, including a decrease in *Lactobacillus*, an increase in parathyroid hyperplasia, and a decrease in intestinal calcium. Fecal microbiota transplantation and *L. johnsonii* colonization indicate a link between gut microbes and CKD–SHPT. Clinically, higher *L. johnsonii* levels are correlated with milder hyperparathyroidism CKD–SHPT. The receiver operating characteristic (ROC) curve for *L. johnsonii* abundance and surgical risk is 0.81, with the calibration curve confirming predictive accuracy, and decision curve analysis revealing good clinical applicability. In vivo and in vitro experiments show that cyclo(pro‐trp) enhance calcium inflow and lower iPTH levels in intestinal epithelial cells via a calcium‐sensing receptor and transient receptor potential vanilloid 4 pathways. This study identified the crucial role of *L. johnsonii* in CKD–SHPT, unveiling a new mechanism for calcium imbalance and offering novel strategies for SHPT treatment and drug development.

## Graphical abstract

The mechanism map of how *L.johnsonii* generates cyclo(pro‐trp) to alleviate CKD–SHPT by promoting the expression of CaSR and TRPV4 in IECs, enhancing Ca^2+^ absorption, and negatively feedback inhibiting iPTH secretion.

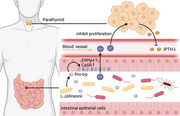

## 1 Introduction

Secondary hyperparathyroidism (SHPT) is a common complication of chronic kidney disease (CKD), and is characterized by increased synthesis and secretion of intact parathyroid hormone (iPTH) and proliferation of parathyroid glands (PTGs).^[^ ^1^, ^2^, ^3^ ^]^ SHPT can occur throughout the course of CKD. The incidence of SHPT in the early stage of CKD is 15%, which increases to 80% in the late stage.^[^ ^4^, ^5^, ^6^ ^]^ SHPT significantly disrupts calcium and phosphorus homeostasis, resulting in serious chronic sequelae such as abnormal bone metabolism and cardiovascular diseases, and ultimately contributing to functional damage across multiple organ systems and increasing patient mortality.^[^ ^7^, ^8^ ^]^ Currently, clarifying the mechanism underlying SHPT and identifying novel effective approaches for the prevention and treatment of SHPT are deemed necessary.

Calcium can only be obtained through dietary sources.^[^ ^9^, ^10^ ^]^ The disturbance of intestinal calcium absorption significantly influences blood calcium levels and modulates iPTH secretion, thereby contributing to SHPT development.^[^ ^10^, ^11^, ^12^ ^]^ Furthermore, the primary pharmacological interventions for SHPT currently employed in clinical practice involve supplementation of active vitamin D, calcium, and calcimimetics to enhance calcium absorption. However, the effect of this supplementation was minimal and could not effectively reduce iPTH levels to prevent SHPT.^[^ ^2^, ^13^ ^]^ Thus, investigating the mechanisms underlying intestinal calcium absorption and developing therapeutic strategies to enhance this process are essential for the treatment of SHPT.

Gut microbes play crucial roles in maintaining intestinal homeostasis, and mediating substance metabolism, and absorption.^[^ ^14^ ^]^ In particular, metabolites of the microbiota, such as peptides and short‐chain fatty acids (SCFAs), promote the absorption of substances such as calcium through active and passive transport.^[^ ^15^, ^16^, ^17^ ^]^ Calcium is mainly absorbed in the duodenum and the region near the jejunum. The disruption of gut microbes can affect calcium absorption by the intestine.^[^ ^18^, ^19^, ^20^, ^21^ ^]^ Recent studies have reported that CKD‐induced gut microbe imbalance can lead to intestinal epithelial cell (IEC) dysfunction, affecting the absorption of substances, particularly calcium.^[^ ^22^, ^23^, ^24^, ^25^ ^]^ However, the relationship between this change and the occurrence of SHPT and the effect on the alteration of iPTH levels, remains unclear.

Based on the abovementioned considerations, we hypothesized that dysbiosis of the gut microbiota plays a role in SHPT onset and progression. Modulating the gut microbiota may augment intestinal calcium absorption, consequently inhibiting iPTH secretion. This could potentially reduce the incidence of SHPT and enhance patient survival rates. This study offers a novel perspective on the underlying mechanisms of the pathogenesis of SHPT and suggests prospective strategies for the prevention and management of CKD–SHPT in the foreseeable future.

## 2 Results

### 2.1 Characteristics of Tissue and Gut Microbiota in Mice with High and Low iPTH Levels

**Figure 1:**
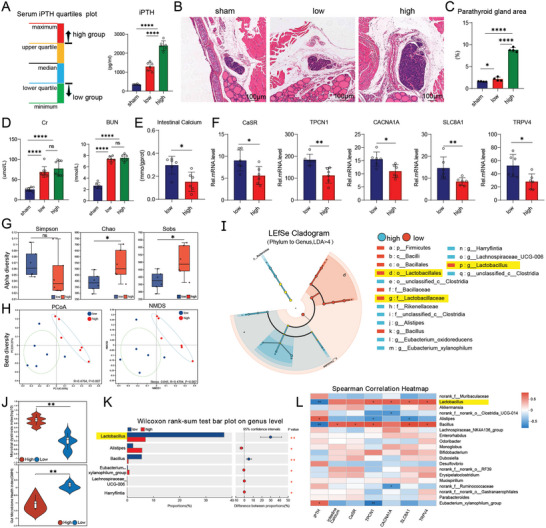
Characteristics of tissue and intestinal microbiota in CKD mouse model. A) Quartile grouping pattern diagram for iPTH in mice, which categorizes them into “high” (iPTH > 2047.7 pg mL^−1^) and “low” (iPTH < 1576.5 pg mL^−1^) groups. Significant differences in iPTH levels were observed among the sham, low, and high groups. B) H&E staining of parathyroid tissues in the sham, low, and high groups. Scale bar = 100 µm. C) Analysis of the parathyroid area scores. The high group exhibited significantly increased parathyroid tissue hyperplasia and area scores, with more severe hyperparathyroidism. D) Serum concentrations of creatinine (CR) and urea nitrogen (BUN) in different mice. Serum CR and BUN levels were significantly higher in the high and low groups than in the sham group, confirming successful CKD modeling. CR and BUN levels were comparable between the high and low groups, suggesting no renal function difference post‐modeling and indicating the influence of other factors on iPTH variations. E) Calcium content of intestine tissue supernatant in different groups, which was significantly increased in the low group compared with that in the high group. F) mRNA expression levels of *CaSR*, *TPCN1*, *CACNA1A*, *SLC8A1*, and *TRPV4* in the intestinal tissue of the low and high groups detected via quantitative polymerase chain reaction (qPCR). The mRNA expression levels were significantly increased in the low group compared with those in the high group. G) Alpha diversity indices at the genus level in CKD–SHPT mouse model. Chao and Sob's analysis showed significant differences. H) Beta diversity indices at the genus levels in CKD–SHPT mouse model (based on PCoA and NMDS). PCoA analysis using Bray–Curtis metric distance algorithms. *R* = 0.4704, *P* = 0.007. NMDS analysis using Bray–Curtis metric distance algorithms. Stress = 0.045, *R* = 0.4704, *P* = 0.007. I) The linear discriminant analysis (LDA) effect size analysis was performed to explore the different species characteristics from the phylum to the genus level between both groups (LDA > 4). The abundance of *Lactobacillus* was higher in the in the low group. J) Microbial dysbiosis index (MDI) and gut microbiota health index (GMHI). MDI testing revealed that higher MDI in the high group and higher GMHI in the low group, indicating closer alignment with healthy population values. K) Gut microbes at the genus levels were analyzed with Wilcoxon rank‐sum test to calculate the P‐value. The high group showed a significant decrease in *Lactobacillus* abundance. L) Spearman correlation heatmap graph of microbiota abundance with iPTH, intestine calcium content, and calcium channel protein expression levels. Results indicated that the *Lactobacillus* strain with the greatest microbiota abundance difference was significantly negatively correlated with iPTH levels and positively correlated with both small intestine calcium content and calcium channel protein expression. The values are presented as mean ± SD in Figure 1A,C–L (*n* = 6) and Figure 1B (*n* = 4); ^*^ *p* < 0.05; ^**^ *p* < 0.01; ^***^ *p* < 0.001; ****p < 0.001 compared with the control group.

Adenine‐containing feed was used to construct a classic mice renal failure model, and mice exhibited differences in iPTH levels. The iPTH levels in mice were defined as “high” (iPTH > 2047.7 pg mL^−1^) and “low” (iPTH < 1576.5 pg mL^−1^) using the quartile method. Compared with the sham group, the high and low group iPTH were significantly elevated. Compared with the high group, the iPTH levels in the low group significantly reduced, showing significant differences (**Figure** **1**A). Compared with the sham group, the low group had increased parathyroid tissue hyperplasia and area scores, but were not significant. Compared with the low group, the high group had significantly increased parathyroid tissue hyperplasia and area scores, with more severe hyperparathyroidism (Figure 1B,C). Compared with the sham group, the high and low groups had significantly increased CR and BUN levels, indicating that CKD modeling was successful. However, no differences in CR and BUN levels were found between the high and low groups, indicating the lack of between‐group difference in renal function after CKD modeling and the presence of other factors that affect the increase or decrease in iPTH (Figure 1D). Obstruction of intestinal calcium absorption is an important factor that affects blood calcium, and to regulate iPTH‐induced hyperparathyroidism,^[^ ^26^, ^27^, ^28^ ^]^ calcium content in the SI tissue was measured. Compared with the high group, the low group showed a significant increase (Figure 1E). At the same time, we detected mRNA levels of calcium channel proteins related to calcium absorption. Compared with the high group, the low group showed significant increases in *CaSR*, *TPCN1*, *CACNA1A*, *SLC8A1*, and *TRPV4* (Figure 1F).

Numerous studies have shown that the gut microbiota imbalance in patients with CKD is related to the absorption of calcium ions (Ca^2+^) in the intestines.^[^ ^29^, ^30^, ^31^ ^]^ To verify whether changes in gut microbiota are associated with calcium absorption and increased iPTH levels, fecal samples were collected from the high and low groups, and 16S rRNA sequencing was performed. Alpha diversity analysis revealed significant differences in chao and sobs; however, the difference in Simpson was not significant (Figure 1G). Diversity analysis among microbiome samples (β‐diversity) showed significant differences in principal coordinate analysis (PCoA) and non‐metric multidimensional scaling (NMDS) (Figure 1H). Linear discriminant analysis (LDA) effect size (LEfSe) analysis (LDA > 4) showed higher abundance levels of *Firmicutes*, *Bacilli*, and *Lactobacillus* in the low group and *Clostridia* and *Rikenellaceae* in the high group (Figure 1I). The microbial dysbiosis index (MDI) test showed higher MDI in the high group, indicating a greater degree of microbial dysbiosis. The gut microbial health index test showed a higher gut microbiota health index in the low group, which is closer to the value for healthy populations (Figure 1J). In addition, a differential test was conducted on the abundance of microbial communities, and compared with the low group, the high group showed a significant decrease in *Lactobacillus* (Figure 1K). To investigate whether the differential changes in microbiota were related to iPTH and intestinal calcium absorption, Spearman correlation analysis was conducted. This analysis revealed that *Lactobacillus*, with the largest difference in microbiota abundance, was negatively correlated with iPTH levels and positively correlated with SI calcium content and calcium channel protein expression, which has statistical significance (Figure 1L).

### 2.2 Gut Microbiota of the Low Group Mice Could Independently Alleviate the Severity of Hyperparathyroidism

**Figure 2:**
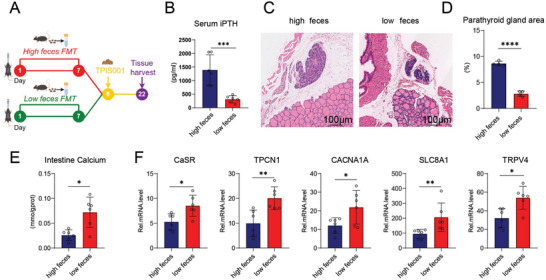
Intestinal microbiota from the low group could independently promote intestinal calcium absorption and further reduce iPTH levels and parathyroid tissue proliferation. A) Flow diagram of the experimental approach: mice were pretreated with high‐group feces FMT and low‐group feces FMT once a day for 7 consecutive days and then administered TPIS001 for 15 days. B) Significant differences in iPTH levels were observed between the high and low feces groups of mice. C) H&E staining of parathyroid tissues in high feces and low feces mice. Scale bar = 100 µm. D) Analysis of parathyroid area scores. The high feces group had significantly increased parathyroid tissue hyperplasia and area scores, with more severe hyperparathyroidism. E) Calcium content of the intestine tissue supernatant in different groups, which was significantly increased in the low feces group compared with that in the high feces group. F) mRNA expression levels of *CaSR*, *TPCN1*, *CACNA1A*, *SLC8A1*, and *TRPV4* in the intestinal tissue of different groups detected via qPCR; all levels were significantly increased in the low feces group compared with those in the high feces group. The values are presented as mean ± SD in Figure 2B,E,F (*n* = 6) and Figure 2C,D (*n* = 4); ^*^ *p* < 0.05; ^**^ *p* < 0.01; ^***^ *p* < 0.001; ****p < 0.0001 compared with the control group.

Previous experiments have shown that gut microbiota, particularly *Lactobacillus*, may be associated with SI calcium content and calcium channel protein expression, thereby regulating iPTH and leading to differences in the severity of hyperparathyroidism. FMT experiments were conducted to further demonstrate the important role of gut microbiota in the severity of hyperparathyroidism (**Figure**  **2**A). CKD model mice were constructed after gavage of feces from the high and low groups, and the iPTH levels in the high feces group were significantly increased compared with those in the low feces group mice (Figure 2B). Compared with the low feces group mice, the high feces group showed significant increases in parathyroid tissue hyperplasia and area scores (Figure 2C,D). The intestinal tract of mice was collected to measure the calcium content, which was significantly increased in the low feces group compared with that in the high feces group mice (Figure 2E). Moreover, the mRNA expression levels of *CaSR*, *TPCN1*, *CACNA1A*, *SLC8A1*, and *TRPV4* in the low feces group were higher than those in the high feces group (Figure 2F).

### 2.3 *L. johnsonii* Can Alleviate the Severity of Hyperparathyroidism

**Figure 3:**
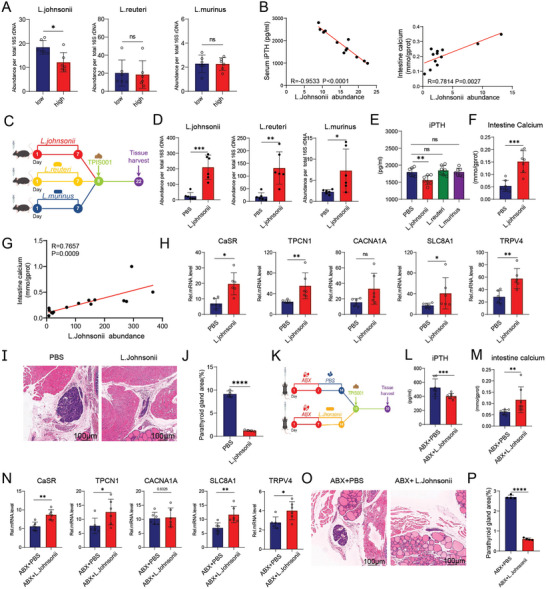
*L. johnsonii* promotes intestinal calcium absorption and further reduces iPTH levels and parathyroid tissue proliferation. A) Relative abundance levels of *L. johnsonii*, *L. reuteri*, and *L. murinus* in the high and low groups, which were significantly increased in the low group compared with those in the high group. B) Correlation linear graph. The relative abundance of *L. johnsonii* was negatively correlated with iPTH levels and positively correlated with small intestinal calcium content. C) Flow diagram of the experimental approach: mice were pretreated with *L. johnsonii*, *L. reuteri*, and *L. murinus* once a day for 7 consecutive days and then administered TPIS001 for 15 days. D) Relative abundance levels of *L. johnsonii*, *L. reuteri*, and *L. murinus* in the feces after colonization. The abundance levels of all three different bacterial strains had increased, proving that strain colonization was successful. E) iPTH levels in mice after colonization with three different bacterial strains. The iPTH levels of mice colonized with *L. johnsonii* were significantly reduced compared with those in mice treated with PBS but were not significantly different in mice inoculated with *L. reuteri* and *L. murinus*. F) Calcium content of intestine tissue supernatant in different groups, which was significantly increased in *L. johnsonii*‐treated mice compared with that in PBS‐treated mice. G) Correlation linear graph. After *L. johnsonii* inoculation, its relative abundance was positively correlated with intestinal calcium content. H) mRNA expression of CaSR, TPCN1, CACNA1A, SLC8A1, and TRPV4 in the intestinal tissues in different groups detected via qPCR, which were all significantly increased in the *L. johnsonii*‐treated mice compared with the mice treated with PBS. I) H&E staining of parathyroid tissue in *L. johnsonii‐* and PBS‐treated mice. Scale bar = 100 µm. J) Analysis of parathyroid area scores. The PBS‐treated mice had significantly increased parathyroid tissue hyperplasia and area scores, with more severe hyperparathyroidism. K) Flow diagram of the experimental approach: mice were pretreated with ABX once a day for 7 consecutive days and then inoculated with *L. johnsonii* once a day for 7 consecutive days starting from day 8, and TPIS001 was administered for 15 days. L) Levels of serum iPTH in mice, which were significantly reduced in the ABX+*L. johnsonii*‐treated mice compared with those in ABX+PBS‐treated mice. M) Calcium content of intestine tissue supernatant in different groups, which was significantly increased in ABX+*L. johnsonii‐*treated mice compared with ABX+PBS‐treated mice. N) mRNA expression levels of CaSR, TPCN1, CACNA1A, SLC8A1, and TRPV4 in intestinal tissue in different groups detected via qPCR. Expression levels of CaSR, TPCN1, SLC8A1, and TRPV4 were significantly increased in ABX+*L. johnsonii‐*treated mice compared with those in ABX+PBS‐treated mice. O) H&E staining of the parathyroid tissues of ABX+*L. johnsonii* and ABX+PBS‐treated mice. Scale bar = 100 µm. P) Analysis of parathyroid area scores. ABX+PBS‐treated mice had significantly increased parathyroid tissue hyperplasia and area score, with more severe hyperparathyroidism. The values are presented as mean ± SD in Figure 3A,B (*n* = 6), Figure 3D–H,L–N (*n* = 6–7), and Figure 3I,J, O,P (*n* = 4); ^*^ *p* < 0.05; ^**^ *p* < 0.01; ^***^ *p* < 0.001; ****p < 0.0001 compared with the control group.

*16S rRNA* sequencing revealed that the relative abundance of *Lactobacillus* in the low group was four times higher than that in the high group (Figure 1K). According to reports, the three common strains of *Lactobacillus* include *L. johnsonii*, *L. reuteri*, and *L. murinus*.^[^ ^32^, ^33^ ^]^ Fecal DNA was extracted for relative abundance detection to further demonstrate the correlation between *L. johnsonii* and iPTH and SI calcium content (**Figure** **3**A,B). This result prompted us to investigate whether oral *L. johnsonii* can alleviate CKD‐induced parathyroid hyperplasia. *L. johnsonii*, *L. reuteri*, and *L. murinus* were cultured, and mice were colonized with a single strain. After 7 days, fecal samples were collected for relative abundance testing. The results showed that after colonization, the abundance of *L. johnsonii*, *L. reuteri*, and *L. murinus* in mice significantly increased, proving that single‐strain colonization was successful (Figure 3C,D). In the constructed CKD mouse models, the iPTH levels of mice colonized with *L. johnsonii* were significantly reduced, whereas those of mice treated with *L. reuteri* and *L. murinus* remained unchanged (Figure 3E). In addition, the SI calcium content in the intestines of *L. johnsonii*‐treated mice increased threefold (Figure 3F). The relative abundance of *L. johnsonii* in mice was significantly positively correlated with the SI calcium content (Figure 3G). Calcium channel proteins related to calcium absorption were evaluated, and *CaSR*, *TPCN1*, *CACNA1A*, *SLC8A1*, and *TRPV4* were found to be significantly elevated in *L. johnsonii*‐treated mice (Figure 3H). Importantly, the proliferation and area scores of the parathyroid tissue in *L. johnsonii*‐treated mice were significantly reduced (Figure 3I,J).

To further validate the direct action of *L. johnsonii* rather than through interaction with other strains in the intestine, mice were pretreated with ABX for 1 week. Quantitative PCR tests confirmed effective bacterial clearance, as shown by the reduced levels of *L. johnsonii*, *L. reuteri*, and *L. murinus* after ABX treatment (Figure S1, Supporting Information). After the gut microbiota was cleared, the mice were treated with *L. johnsonii* for 1 week, and then a CKD mouse model was constructed (Figure 3K). The results showed that compared with the ABX group, the iPTH levels of *L. johnsonii‐*treated mice were significantly reduced (Figure 3L). The SI calcium content in the mice colonized with *L. johnsonii* after ABX administration was two times higher than that in the ABX group (Figure 3M). The mRNA levels of *CaSR*, *TPCN1*, *CACNA1A*, *SLC8A1*, and *TRPV4* in *L. johnsonii‐*treated mice were significantly upregulated (Figure 3N). At the histological level, compared with the ABX mice, the proliferation and area scores of the parathyroid tissue in *L. johnsonii‐*treated mice were significantly reduced after ABX administration (Figure 3O,P). The above results indicate that *L. johnsonii* plays an important role in mediating calcium absorption in the SI and alleviating the severity of hyperparathyroidism in CKD.

### 2.4 *L. johnsonii* Abundance Is Associated with Poor Prognosis in Patients with Hyperparathyroidism

**Figure 4:**
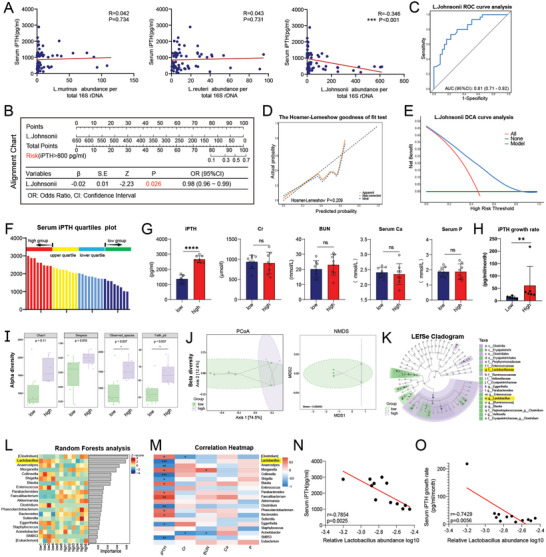
Characteristics of gut microbes in SHPT. A) Correlation linear graph. The PTH levels in patients with hyperparathyroidism is negatively correlated with the relative abundance of *L. johnsonii* but not with the abundance of *L. reuteri* or *L. murinus*. B) Nomogram of the relative abundance of *L. johnsonii* in predicting the probability of individual surgical risk, exhibiting statistical significance. C) Receiver operating characteristic (ROC) curve analysis of the nomograms. The area under the curve corresponding to the abundance of *L. johnsonii* was 0.81 (95% CI, 0.71–0.92). D) Calibration curves of the nomogram. The P‐value of the HL test was 0.209, greater than 0.05, and no significant difference was found between the predicted and true values. E) Decision curve analysis of the nomogram. The net benefit of the model was higher than the All lines and None lines. The abundance of *L. johnsonii* can predict the need for surgery. F) Quartile grouping pattern diagram for iPTH in patients with hyperparathyroidism. G) Serum levels of PTH, CR, BUN, Ca, and P in patients with hyperparathyroidism. Serum PTH levels were significantly higher in the high group than in the low group of patients. CR, BUN, Ca, and P levels were comparable between high and low groups of patients, indicating that the difference in PTH levels is not caused by differences in renal failure but by other factors. H) Increase rates of PTH levels in patients with hyperparathyroidism. The high group had a faster increase rate of PTH levels. I) Alpha diversity indices at the genus level in patients with hyperparathyroidism. Observed_species and Faith_pd analysis showed significant differences. J) Beta diversity indices at the genus levels in patients with hyperparathyroidism (based on PCoA and NMDS). PCoA using weighted_unifrac metric distance algorithms. NMDS analysis using weighted_unifrac metric distance algorithms. Stress = 0.000692. K) Linear discriminant analysis (LDA) effect size analysis was performed to explore the different species characteristics from the phylum to the genus level between the two groups (LDA>2). Results showed that the abundance of *Lactobacillus* was higher in the low group of patients. L) Random forest analysis at the genus level. The relative abundance of *Lactobacillus* was an important factor that affected the PTH grouping. M) Spearman correlation heatmap graph of *Lactobacillus* abundance with PTH, CR, BUN, Ca, and P levels. Results indicated that the *Lactobacillus* strain with the greatest microbiota abundance difference was significantly negatively correlated with PTH levels. N) Correlation linear graph. PTH levels were significantly negatively correlated with relative *Lactobacillus* abundance log_10_. O) Correlation linear graph. The increased rates of PTH were significantly negatively correlated with relative *Lactobacillus* abundance log_10_. Figure 4A–E (*n* = 65), Figure 4F (*n* = 32), Figure 4G (*n* = 8), and Figure 4H–O (*n* = 6); ^*^ *p* < 0.05; ^**^ *p* < 0.01; ^***^ *p* < 0.001; ****p < 0.0001.

To further validate the role of *L. johnsonii*, 65 patients with hyperparathyroidism caused by CKD were enrolled. Serum and fecal samples were collected and examined, and the results revealed that the abundance of *L. johnsonii* was related to the severity of hyperparathyroidism. That is, the higher the abundance of *L. johnsonii*, the milder the severity of hyperparathyroidism, consistent with the results in mice (**Figure** **4**A). Meanwhile, *L. reuteri* and *L. murinus* did not affect iPTH levels. Further analysis revealed that the abundance of *L. johnsonii* was related to the surgical risk of patients. Using a nomogram, the probability of patients undergoing surgical treatment in the later stage based on the specific abundance value of *L. johnsonii* was predicted, which showed significance (Figure 4B). In addition, based on the binary logistic regression analysis model, the abundance of *L. johnsonii* was used as the examined variable, and surgical risk as a state variable to plot the ROC curve. The results showed that the area under the curve corresponding to *L. johnsonii* abundance was 0.81 (95% CI: 0.71‐0.92) (Figure 4C). The nomograms were also calibrated using a calibration curve, and the results showed that the HL test had a *p*‐value of 0.209 (>0.05), indicating the lack of significant difference between the predicted and true values, and thus proving good consistency between the observed and predicted results (Figure 4D). Finally, to evaluate the applicability of the prediction model in clinical practice, a decision curve analysis was conducted. The results showed that within the threshold range, the net benefit of the model was higher than the “All lines” and “None lines,” indicating that *L. johnsonii* abundance can predict surgery, which has actual clinical application value (Figure 4E).

To further investigate the role of *L. johnsonii* in surgical cases, 32 patients undergoing hyperparathyroidism surgery were divided into quartiles based on PTH levels (Figure 4F). The PTH of the patients in the high group was significantly elevated compared with that of patients in the low group. Moreover, no significant difference in serum CR, BUN, Ca, and P was found between the two groups (Figure 4G), indicating that the differences in PTH levels were not caused by differences in renal failure. Interestingly, a significant difference was observed in the increased rates of PTH between the two groups of patients before surgery, with the high group of patients showing faster increases than the low group (Figure 4H). Fecal samples were collected from both the high and low groups of patients before surgery, and 16S rRNA sequencing was performed. The alpha diversity analysis revealed that Observed_species and Faith_pd exhibited differences, and the difference in Simpson and Chao was not significant (Figure 4I). Beta diversity analysis showed that PCoA and NMDS demonstrated significant differences (Figure 4J). The LEfSe analysis results (LDA>2) showed that the abundance of *Lactobacillus* was higher in the low group of patients and showed significant differences (Figure 4K). Meanwhile, random forest analysis revealed that *Lactobacillus* was an important factor affecting PTH grouping (Figure 4L). Finally, a correlation analysis revealed a significant negative correlation between PTH levels and *Lactobacillus* abundance (Figure 4M,N). PTH level increase rates were significantly negatively correlated with *Lactobacillus* abundance (Figure 4O).

### 2.5 *L. johnsonii* Supernatant Can Reduce iPTH Levels

**Figure 5:**
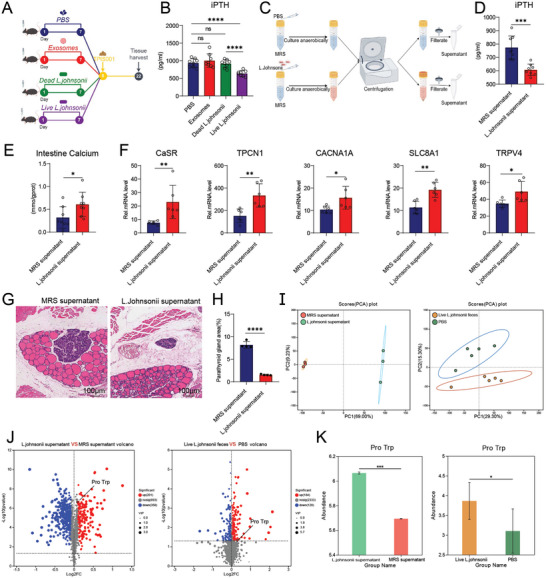
A culture supernatant of *L. johnsonii* can alleviate the severity of hyperparathyroidism. A) Flow diagram of the experimental approach. Mice were pretreated with *L. johnsonii* exosomes, pasteurized *L. johnsonii*, and live *L. johnsonii* once a day for 7 consecutive days and then administered TPIS001 for 15 days. B) iPTH levels in mice. The iPTH levels of mice treated with the live *L. johnsonii* were significantly reduced compared with those of mice treated with PBS. C) Flow diagram of the experimental approach. *L. johnsonii* was cultivated in vitro, and the supernatant of the bacterial solution was collected, purified via ultracentrifugation, and filtered to obtain small molecule metabolites. D) iPTH levels in mice. The iPTH levels in *L. johnsonii* supernatant‐treated mice were significantly reduced compared with those in MRS‐treated mice. E) Calcium contents of intestine tissue supernatant in different groups, which were significantly increased in *L. johnsonii* supernatant‐treated mice compare with those in MRS supernatant‐treated mice. F) mRNA expression levels of *CaSR*, *TPCN1*, *CACNA1A*, *SLC8A1*, and *TRPV4* in the intestinal tissue of mice treated with MRS and *L. johnsonii* supernatant detected via qPCR. Their expression levels were significantly increased in *L. johnsonii* supernatant‐treated mice compared with those in MRS supernatant‐treated mice. G) H&E staining of the parathyroid tissue in MRS‐ and *L. johnsonii* supernatant‐treated mice. Scale bar = 100 µm. H) Analysis of parathyroid area scores. MRS supernatant‐treated mice had significantly increased parathyroid tissue hyperplasia and area scores, with more severe hyperparathyroidism. I) Principal component analysis in different groups. Significant differences were found between MRS supernatant and *L. johnsonii* culture supernatant, as well as gut microbiota (feces) metabolites of mice before and after oral colonization with *L. johnsonii*. J) Volcano plot for metabolites in different groups. Cyclo(pro‐trp) contents in the fecal samples of mice after oral colonization with *L. johnsonii* and treatment with *L. johnsonii* culture supernatant significantly increased. K) Concentrations of cyclo(pro‐trp) in different groups, which were significantly increased in fecal samples of *L. johnsonii* culture supernatant‐treated mice compared with those in the control group. The values are presented as mean ± SD in Figure 5B,D–F (*n* = 6–8), Figure 5G,H (*n* = 4), and Figure 5I–K (*n* = 3–5); ^*^ *p* < 0.05; ^**^ *p* < 0.01; ^***^ *p* < 0.001 compared with the control group.

To further explore what components of *L. johnsonii* reduce iPTH levels, *L. johnsonii* collected from extracellular vesicles, pasteurized *L. johnsonii* bacterial cells, and live *L. johnsonii* bacteria were cultured in vitro, and CKD models were constructed by separately colonizing mice (**Figure** **5**A). Compared with the PBS group of mice, no change in iPTH levels was found in mice orally treated with *L. johnsonii* exosomes and pasteurized *L. johnsonii*; however, the iPTH levels were significantly reduced in mice treated with live *L. johnsonii* (Figure 5B). These results indicated that live *L. johnsonii* bacteria play a role in reducing iPTH levels.

Studies have shown that bacterial metabolites play an important role in influencing the physiological functions of the body.^[^ ^14^ ^]^ *L. johnsonii* may play a role in reducing iPTH levels through its metabolites. To verify this hypothesis, *L. johnsonii* was cultured in vitro, and collected bacterial supernatant was purified via ultracentrifugation to leave behind small molecule metabolites and then administered to mice (Figure 5C). After constructing the CKD mouse model, mouse serum was collected, and iPTH levels were measured. The results showed that compared with mice orally administered with MRS supernatant, those orally administred with *L. johnsonii* culture supernatant showed significantly reduced iPTH levels (Figure 5D). Compared with mice orally administered with MRS supernatant, mice orally administered with *L. johnsonii* culture supernatant had two times increased calcium content (Figure 5E). The expression levels of the calcium channel proteins *CaSR*, *TRPV4*, *TPCN1*, *CACNA1A*, and *SLC8A1* were significantly increased after oral administration of *L. johnsonii* culture supernatant (Figure 5F). At the histological level, compared with mice orally administered with MRS supernatant, those orally administered with *L. johnsonii* culture supernatant had significantly reduced parathyroid tissue hyperplasia and area scores (Figure 5G,H).

To identify differential metabolites, we conducted untargeted metabolomics analysis on MRS supernatant, *L. johnsonii* culture supernatant, and gut microbiota (feces) of mice before and after oral colonization with *L. johnsonii* live bacteria. Principal component analysis revealed significant differences between the MRS supernatant and *L. johnsonii* culture supernatant groups and between before and after oral colonization with live *L. johnsonii* bacteria (Figure 5I). Using Venn diagram screen common metabolites between among them (Figure S2, Supporting Information). Volcanic diagram analysis revealed that the content of cyclo(pro‐trp) in the feces of mice after oral colonization with live *L. johnsonii* bacteria and the supernatant of *L. johnsonii* culture increased, with significant significance (Figure 5J,K). Further, targeted metabolomics analysis was warranted to verify significantly higher cyclo(pro‐trp) levels in the feces of mice implanted with live *L.johnsonii* compared with PBS mice (Figure S3A,B, Supporting Information). At the same time, targeted metabolomics analysis of the feces of SHPT patients revealed that compared with the high group, the low group had significantly higher levels of cyclo(pro‐trp) (Figure S3C,D, Supporting Information). These results indicate that *L. johnsonii* may exert its effects through metabolite cyclo(pro‐trp).

### 2.6 Cyclo(pro‐trp) Can Promote Intestinal Calcium Absorption and Reduce iPTH Levels

**Figure 6:**
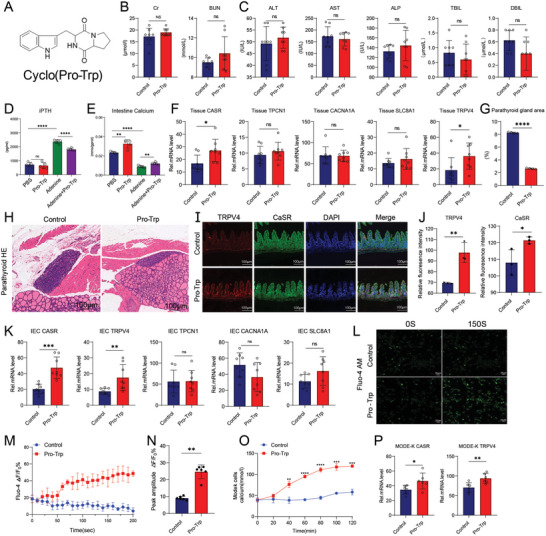
Cyclo(pro‐trp) can alleviate the severity of hyperparathyroidism. A) Molecular structure of cyclo(pro‐trp). B) Serum CR and BUN levels. No damage to renal function. C) Serum ALT, AST, ALP, TBIL, and DBIL levels. No damage to liver function. D) iPTH levels in different groups of mice. Cyclo(pro‐trp) can reduce iPTH levels in CKD mice. E) Calcium contents of intestine tissue supernatant‐treated mice. Cyclo(pro‐trp) can increase the intestinal calcium content before and after CKD modeling in mice. F) mRNA expression levels of *CaSR*, *TPCN1*, *CACNA1A*, *SLC8A1*, and *TRPV4* in intestine tissue of recipient mice. *CaSR* and *TRPV4* significantly increased in cyclo(pro‐trp)‐treated mice. G) Analysis of parathyroid area scores. The control mice had significantly increased parathyroid tissue hyperplasia and area score, with more severe hyperparathyroidism. H) H&E staining of the parathyroid tissue of recipient mice. Scale bar = 100 µm. I) Co‐localization of CaSR and TRPV4 in intestinal epithelial cells (IECs) detected via immunofluorescence staining. Scale bar = 100 µm. J) Fluorescence quantitation in IECs of recipient mice. Significant expression of CaSR and TRPV4 in cyclo(pro‐trp)‐treated mice. K) mRNA expression levels of *CaSR*, *TPCN1*, *CACNA1A, SLC8A1*, and *TRPV4* in the IECs of recipient mice. The expression levels of *CaSR* and *TRPV4* significantly increased in cyclo(pro‐trp)‐treated mice. L) Representative Ca^2+^ imaging of MODE‐K cells. Scale bar = 20 µm. M) Time‐lapse averaged Ca^2+^ traces of MODE‐K cells followed by the administration of cyclo(pro‐trp) (100 µM) in the 10th second after boarding the machine. N) ΔF/F_0_ peak amplitude. The cyclo(pro‐tp) group had significantly higher values than the control group. By unpaired two‐tailed Student's *t*‐test. O) Accumulated bound calcium content in MODE‐K cells. P) mRNA expression levels of *CaSR* and *TRPV4* in MODE‐K cells. The values are presented as mean ± SD in Figure 6B–F,K (*n* = 6–8), Figure 6G,H (*n* = 4), Figure 6I,J (*n* = 3), and Figure 6L,P (*n* = 6); ^*^ *p* < 0.05; ^**^ *p* < 0.01; ^***^ *p* < 0.001 compared with the control group.

Cyclo(pro‐trp) is a stable compound that connects proline and tryptophan by forming a lactam bond, a cyclic molecular structure. Furthermore, it can protect cells from oxidative stress and inflammatory damage.^[^ ^34^ ^]^ Cyclo(pro‐trp) affects the activity of the heart and Ca^2+^ channels, demonstrating antiarrhythmic effects.^[^ ^35^ ^]^ Plant extract cyclo(pro‐trp) was used to explore its effects on iPTH levels and SI calcium absorption, with a chemical molecular formula shown in **Figure** **6**A. According to the method described in the literature, mice were orally administered with 0.5 mg kg^−1^ of cyclo(pro‐trp) for 1 week.^[^ ^36^ ^]^ Then, mice serum was collected to detect CR and BUN levels as renal function indicators and ALT, AST, ALP, TBIL, and DBIL levels as liver function indicators. The results showed no differences in liver and kidney functions between the PBS and cyclo(pro‐trp) groups (Figure 6B,C). This indicates that cyclo(pro‐trp) is nontoxic to the liver and kidney and that it does not affect CKD modeling. In the CKD model, cyclo(pro‐trp) reduced the iPTH level (Figure 6D). Moreover, the SI calcium content was measured, and compared with the PBS group, the cyclo(pro‐trp) group without CKD modeling showed significantly increased SI calcium content. After CKD modeling, cyclo(pro‐trp) continued to enhance the SI calcium content. (Figure 6E). Furthermore, calcium channel proteins related to calcium absorption were detected, and oral administration of cyclo(pro‐trp) in mice significantly increased *CaSR* and *TRPV4* levels; however, no significant difference in *TPCN1*, *CACNA1A*, and *SLC8A1* levels was noted (Figure 6F). Moreover, at the histological level, the proliferation and area scores of parathyroid tissues in mice orally treated with cyclo(pro‐trp) were significantly reduced (Figure 6G,H). In addition, immunofluorescence was used for localization, and the expression levels of CaSR and TRPV4 in epithelial cells increased after oral administration of cyclo(pro‐trp) (Figure 6I,J). Furthermore, extracted primary epithelial cells of the SI were examined for validation, and only *CaSR* and *TRPV4* were upregulated in the primary cells, consistent with histological results (Figure 6K).

To further explore how cyclo(pro‐trp) affects calcium absorption by epithelial cells, mice IEC MODE‐K cells were cultured in vitro. Using Fluo‐4 AM to monitor transient [Ca^2+^]_i_ in MODE‐K cells, cyclo(pro‐trp) was found to promote Ca^2+^ influx in MODE‐K cells. In MODE‐K cells added with cyclo(pro‐trp) in the 10th second, [Ca^2+^]_i_ rapidly increased at 50th second, entered a plateau at 70th second, and remained dynamically stable at 150th second, with significant differences compared with the control group (Figure 6L–N). Finally, the cumulative calcium content of MODE‐K cells was detected, and the results showed that after 20 min of cyclo(pro‐trp) treatment, the calcium content of MODE‐K cells significantly increased and stabilized after 80 min. Compared with the control group, the calcium content of MODE‐K cells increased by 2.1 times after cyclo(pro‐trp) treatment (Figure 6O). Moreover, CaSR and TRPV4 calcium ion channels were significantly upregulated in MODE‐K cells after cyclo(pro‐trp) treatment (Figure 6P).

### 2.7 CaSR and TRPV4 Calcium Channel Inhibition Leads to the Disappearance of the Cyclo(pro‐trp) Effect

**Figure 7:**
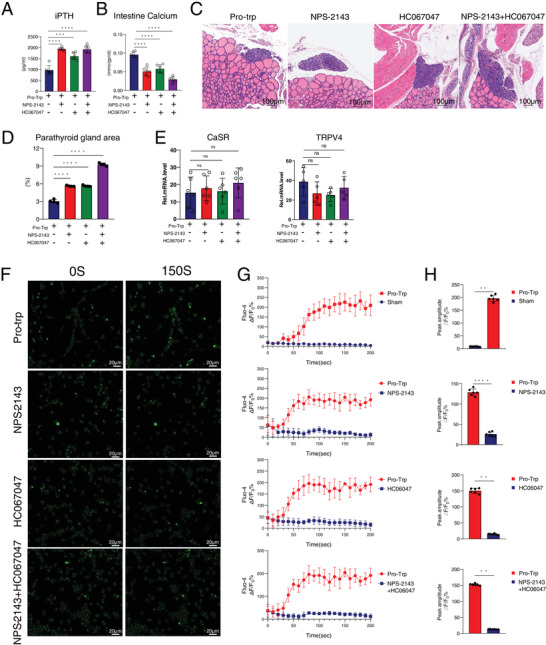
Pharmacological inhibition of CaSR and TRPV4 suppresses cyclo(pro‐trp) effect. A) iPTH levels in different groups of mice. NPS‐2143 and HC067047 can counteract the effect of cyclo(pro‐trp) and increase iPTH levels. B) Calcium content of the intestine tissue supernatant in these mice. NPS‐2143 and HC067047 can counteract the effects of cyclo(pro‐tp) and reduce intestinal calcium contents. C) H&E staining of the parathyroid tissue in these mice. Scale bar = 100 µm. D) Analysis of the parathyroid area scores. The use of NPS‐2143 and HC067047 significantly increased parathyroid tissue hyperplasia and area score, with more severe hyperparathyroidism. E) mRNA expression levels of CaSR and TRPV4 in these mice. F) Representative Ca^2+^ imaging of MODE‐K cells. Scale bar = 20 µm. G) Time‐lapse averaged Ca^2+^ traces of MODE‐K cell pretreated with or without NPS‐2143 and HC067047 for 30 min and administered cyclo(pro‐trp) in the 10th second after boarding the machine. H) ΔF/F_0_ peak amplitude. NPS‐2143 and HC067047 can counteract the effect of cyclo(pro‐trp). By unpaired two‐tailed Student's *t*‐test. The values are presented as mean ± SD in Figure 7A,B,E (*n* = 6), Figure 7C,D (*n* = 4), and Figure 7F–H (*n* = 6); ^*^ *p* < 0.05; ^**^ *p* < 0.01; ^***^ *p* < 0.001; ****p < 0.0001 compared with the control group.

To further validate the effect of cyclo(pro‐trp) on CaSR and TRPV4 calcium channels, the CaSR receptor inhibitor NPS‐2143 and the TRPV4 receptor inhibitor HC067047 were used. CKD model mice were intraperitoneally injected with 3 mg kg^−1^ NPS‐2143 and 1 mg kg^−1^ HC067047. Compared with the cyclo(pro‐trp) group, the iPTH level in the NPS‐2143 group increased by onefold, that in the HC067047 group increased by 0.65‐fold, and that in the NPS‐2143 + HC067047 group by 0.98‐fold (**Figure** **7**A). The SI calcium content test showed that compared with the cyclo(pro‐trp) group, the NPS‐2143 group had a 0.88‐fold decrease, the HC067047 group had a 0.66‐fold decrease, and the NPS‐2143 + HC067047 group had a 2.1‐fold decrease (Figure 7B). At the histological level, the NPS‐2143, HC067047, and NPS‐2143 + HC067047 groups showed significantly increased parathyroid tissue proliferation and area scores (Figure 7C,D).

Furthermore, MODE‐K cell validation was performed in vitro. Compared with the cyclo(pro‐trp) group, MODE‐K cells treated with NPS‐2143, HC067047, and cyclo(pro‐trp) showed no changes in the mRNA levels of *CaSR* and *TRPV4* (Figure 7E), indicating that the use of calcium channel inhibitors did not affect the expression of calcium channels. To further verify the effect of calcium channel inhibitors on cyclo(pro‐trp)‐mediated Ca^2+^ influx in MODE‐K cells, MODE‐K cells were precultured with three combinations of inhibitors: NPS‐2143, HC067047, and NPS‐2143+HC067047. Then, the Fluo‐4 AM indicator was used to monitor [Ca^2+^]_i_ in MODE‐K cells before and after cyclo(pro‐trp) use. Compared with the control group without calcium channel inhibitors, the [Ca^2+^]_i_ changes in MODE‐K cells after using the three combinations of inhibitors disappeared, indicating that NPS‐2143 and HC067047 can inhibit cyclo(pro‐trp)‐mediated Ca^2+^ influx (Figure 7F–H). The above results demonstrate that cyclo(pro‐trp) promotes small intestinal calcium absorption through CaSR and TRPV4.

## 3 Discussion

This study revealed that CKD–SHPT is related to the gut microbiota and that regulating the gut microbiota can alleviate the severity of SHPT. The relative abundance of *L. johnsonii* was negatively correlated with the iPTH levels and positively correlated with the SI calcium content and calcium channel protein expression level. We proved that the live *L. johnsonii* and its metabolites can promote intestinal calcium absorption to reduce iPTH levels, decrease PTG proliferation, and alleviate SHPT severity. Furthermore, the key metabolite cyclo(pro‐trp) was identified using metabolomics, and it was observed that cyclo(pro‐trp) played a critical role in promoting intestinal calcium absorption, reducing iPTH, and alleviating SHPT severity. Importantly, this study revealed that *L. johnsonii* can promote the expression levels of CaSR and TRPV4 in IECs through its metabolite cyclo(pro‐trp), thereby promoting intestinal calcium absorption, which has been further confirmed in in vivo and in vitro experiments. This study indicates that *L. johnsonii* mainly alleviates SHPT by promoting intestinal calcium ion absorption through its metabolite—cyclo(pro‐trp).

The prevalence of SHPT (PTH>300 pg/mL) in the entire European dialysis population ranges from 30% to 49%, with 54% in Americans and 10%–30% in Asians.^[^ ^4^, ^6^, ^8^, ^37^ ^]^ Furthermore, high serum PTH levels and abnormal calcium homeostasis have been reported to increase the risk of cardiovascular disease and fractures in patients,^[^ ^38^, ^39^ ^]^ seriously endangering patients’ quality of life and survival. The pathogenesis of CKD–SHPT is multifactorial, including hypocalcemia, hyperphosphatemia, and low levels of 1,25‐dihydroxyvitamin D (1,25D), all of which can lead to SHPT development in patients with uremia.^[^ ^40^, ^41^, ^42^ ^]^ Numerous studies have shown that SHPT is an important factor in the systemic complex mineral metabolism disorder caused by CKD, in which calcium plays a crucial role.^[^ ^43^, ^44^ ^]^ Therefore, it is worth exploring the potential mechanisms of intestinal calcium absorption disorders, negative feedback leading to an increase in iPTH secretion, and induction of SHPT under the CKD background is worthy. This can further aid in the development of new preventive strategies to reduce the occurrence of SHPT.

At present, SHPT prevention and treatment in clinical practice are limited to internal medicine drug therapy, such as supplementing active vitamin D drugs (1,25D, calcitriol, paricalcitol, and alfacalcidol), calcium, or calcium mimetics (cinacalcet, etelcalcetide, and evocalcet). The main purpose of this therapy is to directly supplement calcium or promote intestinal absorption of calcium; however, these drugs not only induce gastrointestinal symptoms and adverse reactions such as drug interactions but also have poor efficacy, and inhibiting the progression of hyperparathyroidism is difficult.^[^ ^45^, ^46^, ^47^, ^48^, ^49^, ^50^ ^]^ For refractory SHPT, the main treatment methods include US‐guided percutaneous radiofrequency ablation or surgical resection. Although the effect is significant in the short term, it is an invasive treatment with certain surgical risks, and recurrence or triple occurrence is possible.^[^ ^51^, ^52^, ^53^ ^]^ Therefore, exploring new regulatory mechanisms for calcium homeostasis in patients with CKD and developing targeted SHPT prevention and treatment plans are urgent.

The gut microbiota is involved in substance metabolism and absorption, and gut microbiota disruption can affect intestinal homeostasis and substance absorption.^[^ ^14^, ^54^ ^]^ The disruption of gut microbiota homeostasis in patients with CKD interferes with the absorption of minerals such as calcium and phosphorus, leading to an imbalance in the body's calcium homeostasis and further mediating the occurrence and development of hyperparathyroidism.^[^ ^55^, ^56^, ^57^, ^58^, ^59^ ^]^ Therefore, regulating the body's calcium homeostasis by maintaining gut microbiota homeostasis is crucial for CKD–SHPT prevention and treatment. According to reports, synbiotics can improve the intestinal environment, regulate mineral metabolism, and may be one of the treatment options for SHPT. Synbiotics include probiotics and prebiotics produced by their metabolism.^[^ ^60^ ^]^ *Lactobacillus* is one of the main probiotics in the intestines, and various *Lactobacillus* strains have been further characterized as potential probiotics in the food formulation industry. *L. johnsonii* is a novel probiotic and plays an important role in the treatment of various diseases.^[^ ^61^, ^62^, ^63^, ^64^, ^65^ ^]^ Despite the lack of current research on the direct interaction between *L. johnsonii* and SHPT, studies have indicated that FMT with a high abundance of *L. johnsonii* can improve the intestinal microenvironment, regulate microbial amino acid metabolism, and delay CKD progression.^[^ ^66^ ^]^ Planting *L. johnsonii* can increase serum indole‐3‐aldehyde (IAld) levels, inhibit AHR signaling and alleviate CKD.^[^ ^67^ ^]^ Peptides produced by gut probiotics, such as L‐citrulline and its converted L‐arginine, are important mediators of calcium absorption.^[^ ^15^ ^]^ SCFAs such as butyrate and acetate produced by the gut microbiota promote calcium absorption and regulate calcium homeostasis in the body.^[^ ^16^, ^68^, ^69^ ^]^ Based on the above situation, *L. johnsonii* may alleviate CKD–SHPT by promoting calcium absorption through its metabolites.

In this study, we found for the first time that *L. johnsonii* abundance was related to CKD–SHPT development, and the specific abundance value of *L. johnsonii* predicts the risk of patients receiving surgical treatment in the later stage, which has great practical value in clinical applications. Supplementing *L. johnsonii* and its main metabolite cyclo(pro‐trp) was found to alleviate CKD–SHPT, which is undoubtedly a novel finding related to *L. johnsonii*, a probiotic, and has significant implications for CKD–SHPT treatment.

Probiotic metabolites play critical but complex roles in host substance absorption, homeostasis, immunity, infection control, inflammatory disease, and even tumor progression.^[^ ^70^, ^71^ ^]^ In this study, untargeted metabolomics analysis was conducted on mice feces and *L. johnsonii* culture supernatant after oral colonization of live *L. johnsonii*, and the results revealed that cyclo(pro‐trp) content increased. Thus, we speculate that cyclo(pro‐trp) may have played a role as a metabolite of *L. johnsonii*. Cyclo(pro‐trp), as a dipeptide, is one of the simplest peptide derivatives commonly found in nature that exhibit interesting physiological and/or pharmacological activities in mammals.^[^ ^34^ ^]^ Cyclo(pro‐trp), which may exhibit both antimicrobial and antitumor properties, enhances the maturation of the gastrointestinal cells. Therefore, its activity suggests its potential applications as a pharmacological reagent.^[^ ^72^ ^]^ Cyclo(pro‐trp) exerts effects on heart and Ca^2+^ channel activities, demonstrating antiarrhythmic effects and the potential to become a new drug for treating cardiovascular dysfunction.^[^ ^35^ ^]^ This study explored the role of cyclo(pro‐trp) in probiotic‐mediated calcium absorption in the intestines.

Previous studies have revealed that calcium is absorbed by IECs.^[^ ^18^, ^19^, ^20^ ^]^ Similar to *L. johnsonii* single‐bacterium colonization, cyclo(pro‐trp) pretreatment significantly increased intestinal calcium content, reduced PTH levels, and alleviated SHPT, further confirming the important protective role of cyclo(pro‐trp). In the present study, CKD mice showed impaired intestinal calcium absorption and reduced intestinal calcium content compared with sham mice. Although oral administration of cyclo(pro‐trp) can increase intestinal calcium content, it is still low compared with the content in sham mice. Thus, other factors may have interfered with cyclo(pro‐trp)‐mediated intestinal calcium absorption. Moreover, the reasons were further explored. MODE‐K cells were cultivated in vitro, and the intensity of cell [Ca^2+^]_i_ with Fluo‐4 was monitored after adding cyclo(pro‐trp), confirming that cyclo(pro‐trp) can promote calcium influx. Although we have confirmed that cyclo(pro‐trp) can promote calcium absorption in IECs, but there is currently no literature reporting on how live *L. johnsonii* produces cyclo(pro‐trp). We speculate that there may be several possible mechanisms involved. One possibility is that live *L. johnsonii* may contain specific genes encoding proteins rich in pro‐trp, which form cyclo(pro‐trp) after degradation. Secondly, there may be some functional microRNAs in live *L. johnsonii* that directly translate or indirectly regulate mRNA to synthesize cyclo(pro‐trp) enriched peptides. We also cannot exclude the possibility that there are translation‐independent pathways that format cyclo(pro‐trp) in live *L. johnsonii*. Therefore, further research is needed to understand the detailed mechanism of how live *L. johnsonii* produces cyclo(pro‐trp).^[^ ^36^ ^]^ Furthermore, the specific mechanism by which *L. johnsonii* participates in calcium absorption in IECs through cyclo(pro‐trp) remains unclear. Therefore, further research is needed on the molecular mechanisms of the interaction between cyclo(pro‐trp) and IECs, as well as other possible mechanisms.

Numerous studies have shown that calcium absorption in the intestine mainly relies on calcium channel proteins, including CaSR, TRPV4, TPCN1, CACNA1A, and SLC8A1, which play a crucial role in calcium homeostasis.^[^ ^73^, ^74^, ^75^, ^76^ ^]^ CaSR is expressed in the epithelial and villous cells of the small intestine, and it plays an important role in regulating calcium homeostasis and osmosis.^[^ ^77^ ^]^ TRPV4 is widely distributed in the body and is crucial for maintaining cellular function by regulating Ca^2+^ concentration.^[^ ^78^ ^]^ Furthermore, various physical and chemical stimuli can activate TRPV4 channels, which can be antagonized by the antagonist HC‐067047.^[^ ^79^ ^]^ Some studies have reported that mammalian two‐pore channels TPCN1 and TPCN2, calcium channel CaV2.1 encoded by *CACNA1A*, and solution carrier family 8 member A1 (SLC8A1) are channels that are permeable to Ca^2+^.^[^ ^80^, ^81^, ^82^, ^83^ ^]^ This finding confirms that multiple calcium ion channels may play a role in calcium absorption in IECs. However, a detailed understanding of the role of calcium ion channels in the pathogenesis of SHPT is lacking. In this study, compared with the control group, the low group mice, the low feces group mice, colonized with *L. johnsonii* mice and mice orally treated with *L. johnsonii* culture supernatant exhibited high expression levels of *CaSR*, *TPCN1*, *CACNA1A*, *SLC8A1*, and *TRPV4* in the SI, which is consistent with the previous speculation that the probiotic *L. johnsonii* promotes intestinal calcium absorption by increasing the expression of calcium channel proteins. However, only *CaSR* and *TRPV4* were significantly elevated in the SI and epithelial cells of mice orally administered with cyclo(pro‐trp), whereas *TPCN1*, *CACNA1A*, and *SLC8A1* showed no significant differences, indicating the involvement of other *L. johnsonii* metabolites. However, our main focus was on the role of cyclo(pro‐trp). The above results indicated that *L. johnsonii* may alleviate SHPT by increasing the expression levels of *CaSR* and *TRPV4* in the SI, thereby promoting intestinal calcium absorption.

This study had several limitations. Although intestinal microbiota may cause differences in calcium absorption in the intestines of patients with CKD, depending on their individual levels, the gut microbiota system is complex, and the synergistic effects between different bacterial strains may affect the intestines. However, no clear evidence suggests that specific bacterial strains are the most beneficial. In this study, only the effects of *Lactobacillus* strains, namely, *L. johnsonii*, *L. murinus*, and *L. reuteri*, on SHPT were explored. In addition, the cellular receptors involved in the interaction between *L. johnsonii* or cyclo(pro‐trp) and IECs are not clearly understood, which may require further research. Moreover, the increased expression of calcium channels induced by *L. johnsonii* colonization or oral feeding of cyclo(pro‐trp) has not been proven to have a direct effect in vivo. CaSR^−/−^and/or TRPV4^−/−^ mice must be employed to confirm their ability to inhibit intestinal calcium absorption and increase PTH levels, exacerbating SHPT. Finally, the number of patients examined was small; thus, a larger cohort is needed.

## 4 Conclusion

Our results suggest that bacterial dysbiosis occurs in CKD–SHPT. The use of live *L. johnsonii* and its metabolite cyclo(pro‐trp) promotes the expression of intestinal calcium channel proteins, CaSR and TRPV4, thereby facilitating intestinal calcium absorption and inhibiting iPTH synthesis and secretion to prevent SHPT. This study reveals a new mechanism of calcium imbalance in CKD–SHPT and proposes that maintaining calcium homeostasis by maintaining intestinal bacterial homeostasis may be a new therapy for preventing and treating SHPT. In the future, *L. johnsonii* is anticipated to be used as an oral probiotic, or its compound cyclo(pro‐trp) will be developed into a new clinical drug, to tackle calcium supplementation issues in CKD patients. This approach seeks to regulate calcium levels, lower iPTH, and prevent SHPT and related complications.

## 5 Experimental Section

### Ethics Statement

All procedures involving human volunteers were approved by the Ethical Committee of Xinqiao Hospital, Army Medical University (Approval number 2024–181‐01), and all participants provided informed consent. All animal experimental procedures were performed in accordance with the guidelines of the National Institutes of Health Guide for the Care and Use of Laboratory Animals and approved by the local Animal Care and Use Committee of the Xinqiao Hospital of the Army Medical University.

### Patient Cohorts

Patients with uremia stage CKD–SHPT (*n* = 65) were recruited in the nephrology department. These patients showed no significant differences in demographic characteristics such as age and sex. The exclusion criteria were as follows: 1) <18 or >60 years old; 2) had undergone abdominal surgery, used antibiotics in the past 3 months, or had a history of parathyroidectomy; 3) had chronic digestive system diseases and diagnosed with or suspected of malignant tumor; 4) had primary hyperparathyroidism and recurrent and tertiary hyperparathyroidism; and 5) treated with calcium mimetics and/or phosphate binders within 2 weeks.

### DNA Extraction from Fecal Samples

DNA from patients and mice feces was extracted by commercial kits (DNS362‐03, Mabio, Guangzhou, China) according to the manufacturer's instructions.

### 16S rRNA Gene Sequencing

One day before constructing the CKD mouse model with adenine, fresh fecal samples were collected using a sterile enzyme free 2 mL EP tube, immediately frozen in liquid nitrogen, and stored in a −80 °C freezer. Fecal DNA purity testing using NanoDrop2000; 1% agarose gel electrophoresis, voltage 5 V cm^−1^, time 20 min was used for DNA integrity detection. The universal amplicon primers (forward primer: 5′‐GTGTGYCAGCMGCCGCGGTAA‐3′; reverse primer: 5′‐CCGGACTACNVGGGTWTCTAAT‐3′) for the V4 region of bacterial 16S rRNA. PCR products were detected by 2% agarose gel electrophoresis. Synergy HTX was used for PCR products to detect and quantify. NEXTFLEX Rapid DNA – Seq Kit was used to build a library. Purified amplicons paired‐end sequenced on an illumina NextSeq 2000 PE300 platform (illumina, San Diego, USA) according to the standard protocols by Majorbio Bio‐Pharm Technology Co. Ltd. (Shanghai, China). Using QIIME2 (version 2020.2) or data extraction, splicing, and analog peer‐to‐peer bioinformatics analysis.^[^ ^84^ ^]^

### Mice

For this study, 8–10‐week‐old specific pathogen‐free male C57BL/6J mice were purchased from GemPharmatech (N000013, Chengdu, China) and kept at the animal center. All mice received humane care with 12‐h light/dark cycles and had free access to food and water. Furthermore, all germ‐free C57BL/6J mice were provided by and kept in the Medical Animal Center of Army Medical University.

### CKD Mice Models

After 7‐day acclimatization, mice were allocated dietary treatments. The adenine diet used in this study comprised 0.3% adenine and 6% casein (Altromin, Lage, Germany), which was previously reported to blunt the taste and smell of adenine.^[^ ^85^ ^]^ To exclude the potential effects of the casein diet on renal function, the control group was fed the same casein diet as the adenine group but without adenine.

### ABX Mice Models

Mice were given antibiotics (ABX) (vancomycin, 100 mg kg^−1^; neomycin sulfate, 200 mg kg^−1^; metronidazole, 200 mg kg^−1^; and ampicillin, 200 mg kg^−1^) intragastrically once a day for 7 consecutive days to deplete the gut microbiota (receptor mice). At the end of the bacterial clearance, quantitative PCR tests were used to determine the total bacteria count in the feces.

### Fecal Microbiota Transplantation (FMT) Experiment

Before the CKD model was constructed, the collected feces of the high and low groups (donor mice) were resuspended in phosphate‐buffered saline (PBS) at 0.125 g mL^−1^, and 0.2 mL of the solution was administered via oral gavage to the indicated mice at 1 week (6–8 mice per group)

### Lactobacillus Culture and Pretreatment Experiment

*Lactobacillus johnsonii* (DSM 100 219), *Lactobacillus reuteri* (DSM 20 016), and *Lactobacillus murinus* (DSM 100 193) were purchased from Beina Biotech and confirmed at the species level via 16S ribosomal RNA sequencing (V4 sequences). Bacteria were cultured in De Man, Rogosa, and Sharpe (MRS) medium (M8330, Solarbio, China) under an atmosphere of 10% H_2_, 10% CO_2_, and 80% N_2_ (AW500SG Anaerobic Workstations; ELECTROTEK, England) for 24 h at 37 °C. The cultures were resuspended at a final concentration of 5 × 10^8^ CFU/200 µL. Then, single strains were administered via oral gavage to the indicated mice once a day for 7 consecutive days.

### Quantitative Analysis of Cyclo(pro‐trp)

For liquid chromatography‐tandem mass spectrometry analysis (UltiMate 3000 (Thermo, USA) and Q EXACTIVE (Thermo, USA)), add 200 µL of acetonitrile to a 2 mL centrifuge tube containing feces, mix well, and sonicate for 20 min. Then the supernatant was collected after centrifuging at 12000 rcf at 4 °C for 5 min. Then 10 µL of each sample was injected into the Acquity UPLC BEH column. Then, the mobile phase included 0.05% Phosphoric acid‐acetonitrile and 100% acetonitrile solution B. The column temperature was kept at 35 °C with a flow rate of 0.3mL min^−1^. For the mass spectrometry process, the ion source type was H‐ESI and the spray voltage was static. The positive ion voltage was 3000 V. Data were analyzed and processed with Thermo Xcalibur (Thermo, USA) software.

### Drug Administration

Mice were treated with 0.5 and 1.5 mg kg^−1^ cyclo(pro‐trp) (CFN90277, ChemFaces, China), and cyclo(pro‐trp) dosage was chosen based on the conversion of the cellular dosage to animal dosage dissolved in 0.1% dimethylsulfoxide (DMSO). Cyclo(pro‐trp) was administered through oral gavage to the indicated mice once a day for 7 consecutive days.

Mice were treated with 3 mg kg^−1^ NPS‐2143 (S2633, Selleck), 1 mg kg^−1^ HC067047 (S6637, Selleck), or its vehicle DMSO intraperitoneally 30 min before cyclo(pro‐trp) was given, with 2‐day intervals for 15 consecutive days. The final concentration of DMSO used in this experiment did not exceed 0.1%, the dose and usage of the aforementioned pharmaceuticals were based on previous studies.^[^ ^86^, ^87^, ^88^ ^]^

### Primary IEC Isolation and Acquisition

Mouse small intestines (SIs) were opened longitudinally with each segment measuring 1.5–2.0 cm and washed with PBS. To dissociate the crypts, tissues were incubated at 37 °C in 2 mM EDTA in PBS for 30 min. Samples were then suspended in PBS and filtered through a 70‐µm cell strainer (BD Falcon). The supernatant was collected, centrifuged at a speed of 600 g for 5 min, and washed in PBS. The IECs of the SIs were then collected.

### Biochemical and Enzyme‐Linked Immunosorbent Assays

Alanine aminotransferase (ALT; C009‐2‐1, Nanjing Jiancheng Bioengineering Institute, Nanjing, China), aspartate aminotransferase (AST; C010‐2‐1, Nanjing Jiancheng Bioengineering Institute, Nanjing, China), alkaline phosphatase (ALP; A059‐1‐1, Nanjing Jiancheng Bioengineering Institute, Nanjing, China), total bilirubin (TBIL; BC5180, Solarbio, Beijing, China), direct bilirubin (DBIL; BC5170, Solarbio, Beijing, China), serum creatinine (CR; C011‐2‐1, Nanjing Jiancheng Bioengineering Institute, Nanjing, China), and serum urea nitrogen (BUN; C013‐2‐1, Nanjing Jiancheng Bioengineering Institute, Nanjing, China) activities in the serum were measured using commercial reagents according to the manufacturer's instructions. Serum iPTH activities in the mice were measured using ELISA kits (D721103‐0096, BBI, China) according to the manufacturers’ instructions.

### Cell Culture

Murine IEC line MODE‐K cells (BNCC338300, BeNa Culture Collection, China) were maintained in RPMI 1640 culture fluid with 10% fetal bovine serum (BI & Viva Cell, China), penicillin (100 U mL^−1^, Sangon Biotech, China), and ciprofloxacin (10 lg mL^−1^, Sangon Biotech). The cells were exposed to 100 µM solutions (pH 7.4) of cyclo(pro‐trp), as previously described.^[^ ^35^ ^]^ The cyclo(pro‐trp) solutions were stored at 4 °C until use. All materials and solvents used were of analytical grade. Cyclo(pro‐trp) was dissolved in DMSO to a final concentration of 0.5% to aid dissolution.

### Measurement of Calcium Levels

SI and MODE‐K cells were homogenized and measured using commercial assay kits for calcium (C004‐2‐1, Nanjing Jiancheng Bioengineering Institute) according to the manufacturer's instructions.

In vitro Ca^2+^ imaging: [Ca^2+^]_i_ was measured using Fluo‐4 AM (HY‐101896, MCE) as previously described.^[^ ^89^ ^]^ MODE‐K cells were cultured in RPMI 1640 medium. One day later, MODE‐K cells were processed for Ca^2+^ measurements. In brief, MODE‐K cells were put on dishes loaded with Fluo‐4 AM (3 µmol L^−1^ reconstituted in DMSO) and 0.1% Pluronic F‐127 (P3000MP, Thermo Fisher Scientific, MA, USA) in Hanks’ balanced salt solution at 37 °C for 1 h. This step should be performed as quickly as possible to avoid decomposition and subsequent loss of cell‐loading capacity. Subsequently, MODE‐K cells were treated with 100 µM cyclo(pro‐trp) or PBS. The measurements were conducted using a confocal microscope (TCS‐SP8, Leica). Time‐lapse images were captured every 10 s. Signals were expressed in Reflectance Fluorescence Units. The recording duration was 200 s. Before Ca^2+^ measurements, 10 µM CaSR receptor inhibitor NPS‐2143 and 3 µM TRPV4 receptor inhibitor HC067047 were added. Images were obtained by time‐series scanning mode and sampled at 1 fps using a confocal laser scanning microscope (Olympus, Japan). The fluorescence intensity was analyzed offline. To standardize the fluorescence intensity of intracellular Ca^2+^ indicators, the change in [Ca^2+^]_i_ was calculated by the equation: Δ[Ca^2+^]_i_ = ΔF/F_0_ = (F  −  F_0_)/F_0_, where F is the fluorescence intensity of MODE‐K cells at any given time and F0 is the basal fluorescence intensity before experimental manipulation.

### Histopathological Examination

Each mouse was bled while under ether anesthesia. For PTG histologic analysis, an en bloc section of the mouse neck, which included the thyroid, PTGs, trachea, and surrounding muscles and soft tissues, was obtained. PTG tissues were fixed in 4% paraformaldehyde (Sangon Biotech, China), as previously described.^[^ ^90^ ^]^ The mean surface area of the PTGs stained with hematoxylin and eosin (H&E) was measured and evaluated by a blinded observer using ImageJ, as previously described.^[^ ^91^ ^]^

### Immunofluorescence Staining

To determine the expression of CaSR, and TRPV4 in IEC, paraffin sections were incubated overnight at 4 °C with anti‐CaSR antibody (1:100; AF6296; Affinity, USA) and anti‐TRPV4 antibody (1:100; ACC‐034; Alomone Labs, Israel) as previously described. Next, the intestinal paraffin slide was washed and stained with DAPI (C1005; Beyotime, China) for 20 min, and images were captured using a laser scanning confocal microscope (Olympus, Japan). Background intensity was subtracted from the images, and a ratio image was calculated using ImageJ. The average intensity of each channel and the ratio were determined.

### PCR Analyses

Total tissue RNA was extracted using a triazole reagent (Takara, Tokyo, Japan). After mixing the cDNA with SYBR Green Master Mix (Promega, Madison, WI, USA) and specific primers, quantitative reverse‐transcription PCR was performed on a 7500 Real‐Time PCR System (Applied Biosystems, MA, USA). Specific primer sequences are listed in Table S1 (Supporting Information).

### Quantification and Statistical Analyses

Measurement data of normal distribution are presented as mean ± standard deviation (SD), whereas those of non‐normal distribution are presented as median (25th–75th percentiles). For human studies, patients’ basic data were compared using paired student's *t* test for normally distributed data or Wilcoxon signed‐rank test for non‐normally distributed data. Patients’ measurement data were analyzed using Zstats1.0 and Rversion 4.4.0. Binary logistics were used as a regression model for univariate analysis, and nomograms were used to evaluate the predictability of the effect of *L. johnsonii* abundance on the risk of CKD–SHPT surgery. The receiver operating characteristic (ROC) curve method was used to evaluate the predictive value of *L. johnsonii* abundance in assessing the risk of CKD–SHPT surgery. Hosmer–Lemeshow (HL) test was used to test the nomogram, and decision curve analysis was used to validate the clinical practical value of the prediction model. For animal studies, significance between two groups was assessed using a two‐tailed unpaired Student's *t* test, whereas multiple sets of data were subjected to analysis via one‐way analysis of variance (ANOVA) with Tukey post‐hoc test (one‐way ANOVA Tukey), and all the analyses were performed using GraphPad Prism 9.0, unless otherwise stated. All data were assessed using normality and log‐normality tests. The statistics of *p*‐values were provided as ^*^ *p* < 0.05, ^**^ *p* < 0.01, and ^***^ *p* < 0.001, and ns indicated no significance.

## Conflict of Interest

The authors declare no conflict of interest.

## Author Contributions

X.Z., L.S., and H.X. contributed equally to this work. S.G., X.Z., Y.C., S.W., and W.X. performed experimental design and manuscript writing. W.X., Y.C., and S. G. performed study supervision. S.W., C.L., and X.Z. performed clinical investigation and sample collection. X.Z., L.S., H.X., and Z.X. performed experiment organization and implementation. H.G., B.H., and W.W. performed experimental proponents. Y.C., Z.X., J.Z., and Z.Z. performed data analysis. All authors have read and approved the final manuscript.

## Supporting information



Supporting Information

## Data Availability

The data that support the findings of this study are available from the corresponding author upon reasonable request.

## References

[1] J. Cunningham , F. Locatelli , M. Rodriguez , Clin. J. Am. Soc. Nephrol. 2011, 6, 913. 21454719 10.2215/CJN.06040710

[2] L. Magagnoli , P. Ciceri , M. Cozzolino , Expert Opin. Investig. Drugs 2024, 33, 775. 10.1080/13543784.2024.2369307 38881200

[3] L. M. McCann , J. Beto , J Ren Nutr 2010, 20, 141. 20303786 10.1053/j.jrn.2010.01.004

[4] E. Hedgeman , L. Lipworth , K. Lowe , R. Saran , T. Do , J. Fryzek , Int J Nephrol 2015, 2015, 184321. 25918645 10.1155/2015/184321 PMC4396737

[5] T. Isakova , P. Wahl , G. S. Vargas , O. M. Gutiérrez , J. Scialla , H. Xie , D. Appleby , L. Nessel , K. Bellovich , J. Chen , L. Hamm , C. Gadegbeku , E. Horwitz , R. R. Townsend , C. A. Anderson , J. P. Lash , C. Y. Hsu , M. B. Leonard , M. Wolf , Kidney Int. 2011, 79, 1370. 21389978 10.1038/ki.2011.47 PMC3134393

[6] Y. Xu , M. Evans , M. Soro , P. Barany , J. J. Carrero , Clin Kidney J. 2021, 14, 2213. 34603697 10.1093/ckj/sfab006 PMC8483675

[7] J. Floege , J. Kim , E. Ireland , C. Chazot , T. Drueke , A. de Francisco , F. Kronenberg , D. Marcelli , J. Passlick‐Deetjen , G. Schernthaner , B. Fouqueray , D. C. Wheeler , Nephrol Dial Transplant 2011, 26, 1948. 20466670 10.1093/ndt/gfq219 PMC3107766

[8] L. Magagnoli , M. Cozzolino , F. J. Caskey , M. Evans , C. Torino , G. Porto , M. Szymczak , M. Krajewska , C. Drechsler , P. Stenvinkel , M. Pippias , F. W. Dekker , E. N. M. de Rooij , C. Wanner , N. C. Chesnaye , K. J. Jager , Nephrol Dial Transplant 2023, 38, 2562. 37230954 10.1093/ndt/gfad100 PMC10615632

[9] E. Torres‐Maravilla , A. S. Boucard , A. H. Mohseni , S. S. Taghinezhad , N. G. Cortes‐Perez , L. G. Bermúdez‐Humarán , Microorganisms 2021, 9. 34068653 10.3390/microorganisms9051021 PMC8151957

[10] S. D. Bianco , J. B. Peng , H. Takanaga , Y. Suzuki , A. Crescenzi , C. H. Kos , L. Zhuang , M. R. Freeman , C. H. Gouveia , J. Wu , H. Luo , T. Mauro , E. M. Brown , M. A. Hediger , J. Bone Miner. Res. 2007, 22, 274. 17129178 10.1359/jbmr.061110 PMC4548943

[11] V. Muppidi , S. R. Meegada , A. Rehman , in StatPearls Publishing Copyright 2024, StatPearls Publishing LLC, Treasure Island, FL 2024.

[12] M. Cozzolino , A. Galassi , F. Conte , M. Mangano , L. Di Lullo , A. Bellasi , Ther Clin Risk Manag 2017, 13, 679. 28615947 10.2147/TCRM.S108490 PMC5461056

[13] F. Llach , F. Velasquez Forero , Am. J. Kidney Dis. 2001, 38, S20. 10.1053/ajkd.2001.28113 11689384

[14] F. Sommer , J. M. Anderson , R. Bharti , J. Raes , P. Rosenstiel , Nat. Rev. Microbiol. 2017, 15, 630. 28626231 10.1038/nrmicro.2017.58

[15] D. Wang , J. Cai , Q. Pei , Z. Yan , F. Zhu , Z. Zhao , R. Liu , X. Guo , T. Sun , J. Liu , Y. Tian , H. Liu , X. Shao , J. Huang , X. Hao , Q. Chang , Z. Luo , D. Jing , Cell Metab. 2024, 36, 1252. 38718794 10.1016/j.cmet.2024.04.004

[16] Y. Che , G. Chen , Q. Guo , Y. Duan , H. Feng , Q. Xia , Hepatology 2023, 78, 88. 36947402 10.1097/HEP.0000000000000047

[17] X. Zhong , F. Zhang , X. Yin , H. Cao , X. Wang , D. Liu , J. Chen , X. Chen , J Microbiol Biotechnol 2021, 31, 765. 34176870 10.4014/jmb.2104.04016 PMC9705830

[18] S. Sugimoto , E. Kobayashi , M. Fujii , Y. Ohta , K. Arai , M. Matano , K. Ishikawa , K. Miyamoto , K. Toshimitsu , S. Takahashi , K. Nanki , Y. Hakamata , T. Kanai , T. Sato , Nature 2021, 592, 99. 33627870 10.1038/s41586-021-03247-2

[19] A. Arnold , E. Dennison , C. S. Kovacs , M. Mannstadt , R. Rizzoli , M. L. Brandi , B. Clarke , R. V. Thakker , Nat. Rev. Endocrinol. 2021, 17, 261. 33727709 10.1038/s41574-021-00477-2

[20] R. S. Sandler , S. Sun , T. O. Keku , J. T. Woosley , C. Anderson , A. F. Peery , A. Fodor , Clin. Transl. Gastroenterol. 2023, 14, e00569. 37377217 10.14309/ctg.0000000000000569 PMC10299767

[21] B. Waclawiková , P. Cesar Telles de Souza , M. Schwalbe , C. G. Neochoritis , W. Hoornenborg , S. A. Nelemans , S. J. Marrink , S. El Aidy , Gut Microbes 2023, 15, 2154544. 36511640 10.1080/19490976.2022.2154544 PMC9754111

[22] K. Skrypnik , J. Suliburska , J. Sci. Food Agric. 2018, 98, 2449. 28991359 10.1002/jsfa.8724

[23] C. Abraham , M. T. Abreu , J. R. Turner , Gastroenterology 2022, 162, 1602. 35149024 10.1053/j.gastro.2021.12.288 PMC9112237

[24] A. Nallu , S. Sharma , A. Ramezani , J. Muralidharan , D. Raj , Transl. Res. 2017, 179, 24. 27187743 10.1016/j.trsl.2016.04.007 PMC5086447

[25] F. Shao , Y. Yao , D. Weng , R. Wang , R. Liu , Y. Zhang , E. Li , M. Wang , Y. Tang , Y. Ding , Y. Xie , Front. Nutr. 2024, 11, 1364841. 38765814 10.3389/fnut.2024.1364841 PMC11099270

[26] N. Matikainen , T. Pekkarinen , E. M. Ryhänen , C. Schalin‐Jäntti , Endocrinol Metab Clin North Am 2021, 50, 575. 34774235 10.1016/j.ecl.2021.07.005

[27] G. Diaz de Barboza , S. Guizzardi , N. Tolosa de Talamoni , World J. Gastroenterol. 2015, 21, 7142. 26109800 10.3748/wjg.v21.i23.7142 PMC4476875

[28] M. D. Walker , E. Shane , JAMA, J. Am. Med. Assoc. 2022, 328, 1624. 10.1001/jama.2022.18331 36282253

[29] V. Douard , A. Asgerally , Y. Sabbagh , S. Sugiura , S. A. Shapses , D. Casirola , R. P. Ferraris , J. Am. Soc. Nephrol. 2010, 21, 261. 19959720 10.1681/ASN.2009080795 PMC2834550

[30] M. Peacock , Clin. J. Am. Soc. Nephrol. 2010, 5, S23. 20089499 10.2215/CJN.05910809

[31] Q. Y. Hao , J. Yan , J. T. Wei , Y. H. Zeng , L. Y. Feng , D. D. Que , S. C. Li , J. B. Guo , Y. Fan , Y. F. Ding , X. L. Zhang , P. Z. Yang , J. W. Gao , Z. H. Li , Gut Microbes 2024, 16, 2351532. 38727248 10.1080/19490976.2024.2351532 PMC11093026

[32] T. L. Montgomery , A. Künstner , J. J. Kennedy , Q. Fang , L. Asarian , R. Culp‐Hill , A. D'Alessandro , C. Teuscher , H. Busch , D. N. Krementsov , Proc Natl Acad Sci 2020, 117, 27516. 33077601 10.1073/pnas.2002817117 PMC7959502

[33] T. L. Montgomery , K. Eckstrom , K. H. Lile , S. Caldwell , E. R. Heney , K. G. Lahue , A. D'Alessandro , M. J. Wargo , D. N. Krementsov , Microbiome 2022, 10, 198. 36419205 10.1186/s40168-022-01408-7 PMC9685921

[34] C. Prasad , Peptides 1995, 16, 151. 7716068 10.1016/0196-9781(94)00017-z

[35] H. Jamie , G. Kilian , K. Dyason , P. J. Milne , J. Pharm. Pharmacol. 2002, 54, 1659. 12542896 10.1211/002235702252

[36] S. Xie , J. Li , F. Lyu , Q. Xiong , P. Gu , Y. Chen , M. Chen , J. Bao , X. Zhang , R. Wei , Y. Deng , H. Wang , Z. Zeng , Z. Chen , Y. Deng , Z. Lian , J. Zhao , W. Gong , Y. Chen , K. X. Liu , Y. Duan , Y. Jiang , H. W. Zhou , P. Chen , Gut 2023, 73, 78. 37553229 10.1136/gutjnl-2023-329996

[37] A. Levin , G. L. Bakris , M. Molitch , M. Smulders , J. Tian , L. A. Williams , D. L. Andress , Kidney Int. 2007, 71, 31. 17091124 10.1038/sj.ki.5002009

[38] A. S. Go , G. M. Chertow , D. Fan , C. E. McCulloch , C. Y. Hsu , N. Engl. J. Med. 2004, 351, 1296. 15385656 10.1056/NEJMoa041031

[39] G. A. Block , P. S. Klassen , J. M. Lazarus , N. Ofsthun , E. G. Lowrie , G. M. Chertow , J. Am. Soc. Nephrol. 2004, 15, 2208. 15284307 10.1097/01.ASN.0000133041.27682.A2

[40] R. Kumar , J. R. Thompson , J. Am. Soc. Nephrol. 2011, 22, 216. 21164021 10.1681/ASN.2010020186 PMC5546216

[41] K. J. Martin , E. A. González , J. Am. Soc. Nephrol. 2007, 18, 875. 17251386 10.1681/ASN.2006070771

[42] W. G. Goodman , L. D. Quarles , Kidney Int. 2008, 74, 276. 17568787 10.1038/sj.ki.5002287

[43] S. M. Moe , T. B. Drüeke , G. A. Block , J. B. Cannata‐Andía , G. J. Elder , M. Fukagawa , V. Jorgetti , M. Ketteler , C. B. Langman , A. Levin , A. M. MacLeod , L. McCann , P. A. McCullough , S. M. Ott , A. Y. M. Wang , J. R. Weisinger , D. C. Wheeler , R. Persson , A. Earley , R. Moorthi , K. Uhlig , Kidney Int Suppl 2009, S1. 10.1038/ki.2009.188 19644521

[44] T. Isakova , T. L. Nickolas , M. Denburg , S. Yarlagadda , D. E. Weiner , O. M. Gutiérrez , V. Bansal , S. E. Rosas , S. Nigwekar , J. Yee , H. Kramer , Kidney Int Suppl. 2017, 7, 1. 10.1053/j.ajkd.2017.07.019 28941764

[45] M. Fukagawa , R. Shimazaki , T. Akizawa , Kidney Int. 2018, 94, 818. 30049473 10.1016/j.kint.2018.05.013

[46] R. Pandey , J. B. Zella , J. G. Zhu , L. A. Plum , M. Clagett‐Dame , W. J. Blaser , W. Bedale , H. F. DeLuca , D. W. Coyne , Drugs R D 2017, 17, 597. 28905271 10.1007/s40268-017-0210-z PMC5694423

[47] H. Koc , H. Hoser , Y. Akdag , C. Kendir , F. F. Ersoy , Int Urol Nephrol 2019, 51, 1261. 31161518 10.1007/s11255-019-02175-5

[48] S. Okuno , M. Inaba , E. Ishimura , S. Nakatani , H. Chou , S. Shoji , T. Yamakawa , Nephron 2019, 142, 106. 30712039 10.1159/000496808

[49] V. Brandenburg , M. Ketteler , Nutrients 2022, 14. 35893866 10.3390/nu14153009 PMC9330693

[50] C. L. Lu , J. F. Shyu , C. C. Wu , C. F. Hung , M. T. Liao , W. C. Liu , C. M. Zheng , Y. C. Hou , Y. F. Lin , K. C. Lu , Nutrients 2018, 10.30577516

[51] T. Hiramitsu , Y. Hasegawa , K. Futamura , M. Okada , N. Goto , S. Narumi , Y. Watarai , Y. Tominaga , T. Ichimori , Front Endocrinol (Lausanne) 2023, 14, 1169793. 37152972 10.3389/fendo.2023.1169793 PMC10159274

[52] W. Yue , T. Jiang , Z. Ai , E. Deng , H. Chai , X. Li , H. He , Z. Zhang , N. Weng , X. Qin , J. Fan , X. Tang , W. Heng , Y. Li , L. Sun , C. Peng , H. Xu , Radiology 2024, 311, e231852. 38625007 10.1148/radiol.231852

[53] L. X. Zhang , B. Zhang , X. Y. Liu , Z. M. Wang , P. Qi , T. Y. Zhang , Q. Zhang , Front Endocrinol (Lausanne) 2022, 13, 1059828. 36561571 10.3389/fendo.2022.1059828 PMC9763452

[54] X. V. Li , I. Leonardi , I. D. Iliev , Immunity 2019, 50, 1365. 31216461 10.1016/j.immuni.2019.05.023 PMC6585451

[55] A. P. Black , L. F. Cardozo , D. Mafra , Ther Apher Dial 2015, 19, 436. 25944654 10.1111/1744-9987.12307

[56] H. Tanaka , H. Komaba , M. Koizumi , T. Kakuta , M. Fukagawa , J Ren Nutr 2012, 22, 98. 22200424 10.1053/j.jrn.2011.10.031

[57] W. H. Huang , C. C. Hung , C. W. Yang , J. Y. Huang , Ther. Apher. Dial. 2012, 16, 361. 22817125 10.1111/j.1744-9987.2012.01068.x

[58] L. Yan , Q. Xiong , Q. Xu , P. Ren , T. Li , H. Cao , F. Shao , Immun. Inflamm. Dis. 2023, 11, e828. 37102663 10.1002/iid3.828 PMC10091369

[59] M. Rodzoń‐Norwicz , S. Norwicz , M. Sowa‐Kućma , A. Gala‐Błądzińska , Endokrynol. Pol. 2023, 74, 490. 37902013 10.5603/ep.95820

[60] Y. Iwashita , M. Ohya , M. Yashiro , T. Sonou , K. Kawakami , Y. Nakashima , T. Yano , Y. Iwashita , T. Mima , S. Negi , K. Kubo , K. Tomoda , T. Odamaki , T. Shigematsu , Am J Nephrol 2018, 47, 325. 29779028 10.1159/000488947

[61] D. J. Jia , Q. W. Wang , Y. Y. Hu , J. M. He , Q. W. Ge , Y. D. Qi , L. Y. Chen , Y. Zhang , L. N. Fan , Y. F. Lin , Y. Sun , Y. Jiang , L. Wang , Y. F. Fang , H. Q. He , X. E. Pi , W. Liu , S. J. Chen , L. J. Wang , Gut Microbes 2022, 14, 2145843. 36398889 10.1080/19490976.2022.2145843 PMC9677986

[62] S. E. McCollum , Y. M. Shah , Cancer Res. 2024, 84, 645. 38437637 10.1158/0008-5472.CAN-23-3871 PMC12176054

[63] Z. Zhang , L. Zhao , J. Wu , Y. Pan , G. Zhao , Z. Li , L. Zhang , Microorganisms 2023, 11. 37894238 10.3390/microorganisms11102580 PMC10609197

[64] V. Liévin‐Le Moal , A. L. Servin , Clin. Microbiol. Rev. 2014, 27, 167. 24696432 10.1128/CMR.00080-13 PMC3993101

[65] B. Thakur , S. Kaur , N. Rani , R. Kaur , S. K. Upadhyay , M. Tripathi , Mol. Biotechnol. 2023. 10.1007/s12033-023-00937-2 37948026

[66] X. Liu , M. Zhang , X. Wang , P. Liu , L. Wang , Y. Li , X. Wang , F. Ren , Front Microbiol 2022, 13, 1037257. 36532422 10.3389/fmicb.2022.1037257 PMC9748282

[67] H. Miao , F. Liu , Y. N. Wang , X. Y. Yu , S. Zhuang , Y. Guo , N. D. Vaziri , S. X. Ma , W. Su , Y. Q. Shang , M. Gao , J. H. Zhang , L. Zhang , Y. Y. Zhao , G. Cao , Signal Transduct Target Ther 2024, 9, 195. 39098923 10.1038/s41392-024-01913-1 PMC11298530

[68] C. M. Whisner , B. R. Martin , C. H. Nakatsu , J. A. Story , C. J. MacDonald‐Clarke , L. D. McCabe , G. P. McCabe , C. M. Weaver , J. Nutr. 2016, 146, 1298. 27281813 10.3945/jn.115.227256

[69] T. Karakan , K. M. Tuohy , G. Janssen‐van Solingen , Front. Nutr. 2021, 8, 672925. 34386514 10.3389/fnut.2021.672925 PMC8353095

[70] L. M. Williams , S. Cao , Pharmacol. Ther. 2024, 256, 108605. 38367866 10.1016/j.pharmthera.2024.108605 PMC10985132

[71] G. Zavišić , S. Ristić , S. Petričević , D. Janković , B. Petković , Foods. 2024, 13. 39200415 10.3390/foods13162487 PMC11353716

[72] M. Graz , A. Hunt , H. Jamie , G. Grant , P. Milne , Pharmazie 1999, 54, 772. 10563376

[73] W. A. Catterall , M. J. Lenaeus , T. M. Gamal El‐Din , Annu. Rev. Pharmacol. Toxicol. 2020, 60, 133. 31537174 10.1146/annurev-pharmtox-010818-021757

[74] R. Xie , J. Xu , Y. Xiao , J. Wu , H. Wan , B. Tang , J. Liu , Y. Fan , S. Wang , Y. Wu , T. X. Dong , M. X. Zhu , J. M. Carethers , H. Dong , S. Yang , Cancer Res. 2017, 77, 6499. 28951460 10.1158/0008-5472.CAN-17-0360

[75] T. Piekut , Y. Y. Wong , S. E. Walker , C. L. Smith , J. Gauberg , A. N. Harracksingh , C. Lowden , B. B. Novogradac , H. M. Cheng , G. E. Spencer , A. Senatore , Genome Biol Evol 2020, 12, 1217. 32413100 10.1093/gbe/evaa097 PMC7456537

[76] G. L. Guo , L. Q. Sun , M. H. Sun , H. M. Xu , J. Cell. Physiol. 2019, 234, 9019. 30378115 10.1002/jcp.27574

[77] J. Geibel , K. Sritharan , R. Geibel , P. Geibel , J. S. Persing , A. Seeger , T. K. Roepke , M. Deichstetter , C. Prinz , S. X. Cheng , D. Martin , S. C. Hebert , Proc. Natl. Acad. Sci. 2006, 103, 9390. 16760252 10.1073/pnas.0602996103 PMC1475505

[78] J. P. White , M. Cibelli , L. Urban , B. Nilius , J. G. McGeown , I. Nagy , Physiol. Rev. 2016, 96, 911. 27252279 10.1152/physrev.00016.2015

[79] R. Sánchez‐Hernández , M. Benítez‐Angeles , A. M. Hernández‐Vega , T. Rosenbaum , Channels (Austin) 2024, 18, 2313323. 38354101 10.1080/19336950.2024.2313323 PMC10868539

[80] S. Patel , B. S. Kilpatrick , Biochim. Biophys. Acta. Mol. Cell Res. 2018, 1865, 1678. 29746898 10.1016/j.bbamcr.2018.05.004 PMC6162333

[81] S. Patel , Sci. Signal 2015, 8, re7. 26152696 10.1126/scisignal.aab3314

[82] M. Kowalska , M. Prendecki , T. Piekut , W. Kozubski , J. Dorszewska , Int. J. Mol. Sci. 2021, 22. 33799975 10.3390/ijms22052688 PMC7962070

[83] C. R. Rose , D. Ziemens , A. Verkhratsky , Cell Calcium 2020, 86, 102154. 31901681 10.1016/j.ceca.2019.102154

[84] M. Hall , R. G. Beiko , Methods Mol. Biol. 2018, 1849, 113. 30298251 10.1007/978-1-4939-8728-3_8

[85] T. Jia , H. Olauson , K. Lindberg , R. Amin , K. Edvardsson , B. Lindholm , G. Andersson , A. Wernerson , Y. Sabbagh , S. Schiavi , T. E. Larsson , BMC Nephrol. 2013, 14, 116. 23718816 10.1186/1471-2369-14-116 PMC3682934

[86] C. Wang , Q. Jia , C. Sun , C. Jing , Biochem. Biophys. Res. Commun. 2020, 530, 651. 32768195 10.1016/j.bbrc.2020.07.081

[87] Y. Zhen , C. Ding , J. Sun , Y. Wang , S. Li , L. Dong , Am. J. Transl. Res. 2016, 8, 911. 27158378 PMC4846935

[88] F. C. Dias , V. S. Alves , D. O. Matias , C. P. Figueiredo , A. L. P. Miranda , G. F. Passos , R. Costa , Eur. J. Pharmacol. 2019, 856, 172408. 31129158 10.1016/j.ejphar.2019.172408

[89] Y. Yang , M. Shi , X. Liu , Q. Zhu , Z. Xu , G. Liu , T. Feng , T. Stewart , J. Zhang , J. Adv. Res. 2024, 62, 187. 37714326 10.1016/j.jare.2023.09.009 PMC11331169

[90] N. Morito , K. Yoh , T. Usui , H. Oishi , M. Ojima , A. Fujita , R. Koshida , H. H. Shawki , M. Hamada , M. Muratani , K. Yamagata , S. Takahashi , Kidney Int. 2018, 93, 54. 28964572 10.1016/j.kint.2017.06.023

[91] A. K. Yazgan , O. Topaloğlu , F. N. Çuhacı , D. Özdemir , A. Alkan , M. Kılıç , R. Ersoy , B. Çakır , Ann. Diagn. Pathol. 2020, 46, 151492. 32302921 10.1016/j.anndiagpath.2020.151492

