# Supplementary material for: Lactobacillus johnsonii Generates Cyclo(pro‐trp) and Promotes Intestinal Ca2+ Absorption to Alleviate CKD–SHPT
Source: Adv Sci (Weinh). 2025 Jan 31;12(16):2414678. doi: 10.1002/advs.202414678 (PMC12021065; doi:10.1002/advs.202414678)
Supplement: Supplementary file 1 — Supporting Information [file ADVS-12-2414678-s001.docx]

**Supplementary Figures**

***
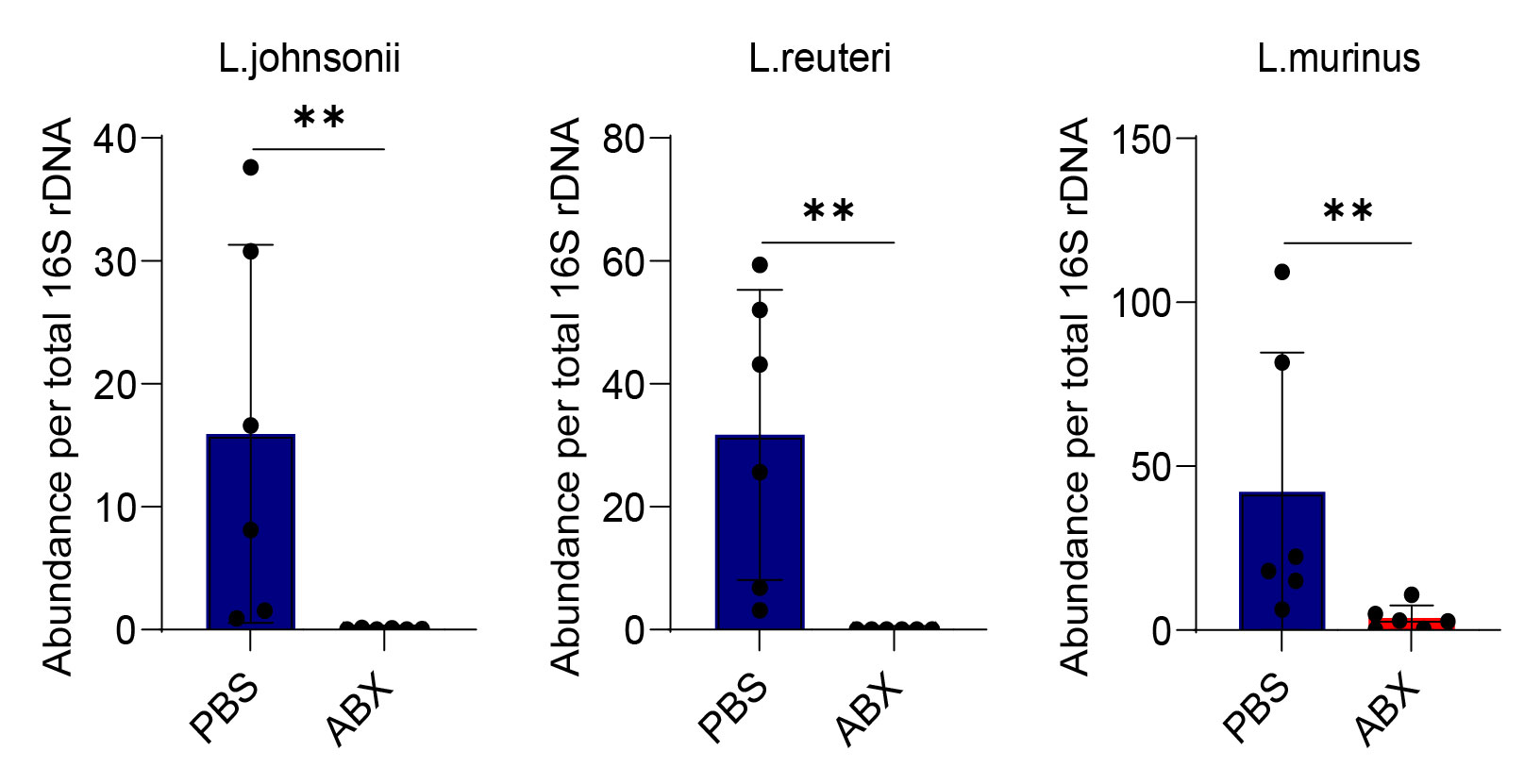
***

**Figure S1.** Relative abundance levels of *L. johnsonii*, *L. reuteri*, and *L. murinus* in the feces after ABX treatment, the abundance levels of all three different bacterial strains decreased, indicating that strains removal was successful. Data were represented as mean ± SEM. Statistical comparison was performed via two-tailed unpaired Student’s t test in Fig S1. *p < 0.05, **p < 0.01.


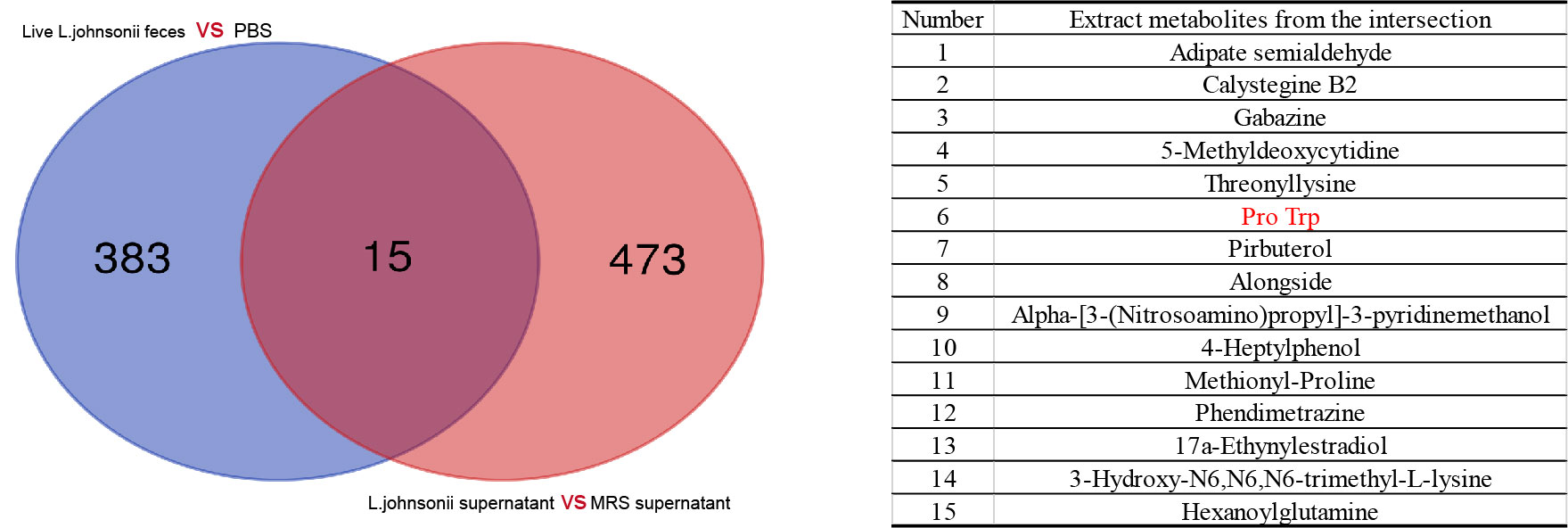


**Figure S2.** A Venn diagram analysis was conducted to compare the fecal differential metabolites with the differential metabolites present in the culture supernatant. This analysis revealed an intersection of 15 metabolites common to both conditions.


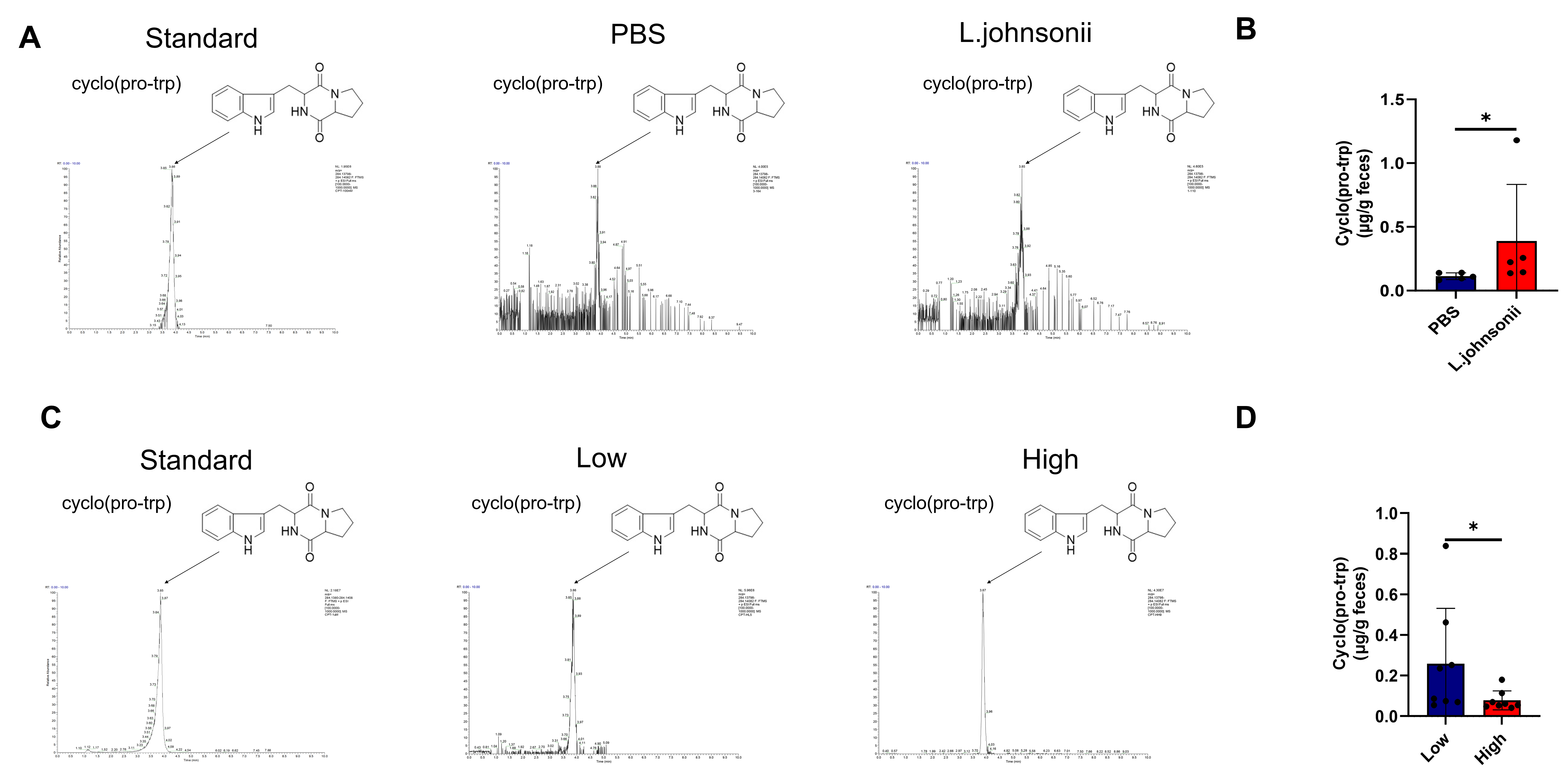


**Figure S3. Concentration of cyclo(pro-trp) in feces.** (A) Mass spectra, targeted metabolomics analysis of cyclo(pro-trp) in cyclo(pro-trp) standard, PBS group feces, and *L .johnsonii* group feces using high-performance liquid chromatography. (B) Concentration of cyclo(pro-trp) in mouse feces, the level of cyclo(pro-trp) in the feces of the *L. johnsonii* group significantly increased (n=5). (C) Mass spectra, targeted metabolomics analysis of cyclo(pro-trp) in cyclo(pro-trp) standard, low feces and high feces of SHPT patients using high-performance liquid chromatography. (D) The concentration of cyclo(pro-trp) in the feces of SHPT patients, the level of cyclo(pro-trp) in the feces of the low significantly increased (n=8). Data were represented as mean ± SD. Statistical comparison was performed via two-tailed unpaired Student’s t test in Fig S3(A-D). *p < 0.05, **p < 0.01, and ***p < 0.001.
